# Supplementary material for: In vivo armed macrophages curb liver metastasis through tumor-reactive T-cell rejuvenation
Source: Nat Commun. 2025 Apr 11;16:3471. doi: 10.1038/s41467-025-58369-2 (PMC11992024; doi:10.1038/s41467-025-58369-2)
Supplement: Supplementary file 1 — Supplementary Information [file 41467_2025_58369_MOESM1_ESM.pdf]

Supplementary Information

In vivo armed macrophages curb liver metastasis through tumor reactive T cell rejuvenation

Marco Notaro [1,2], Maristella Borghetti [1,2], Chiara Bresesti [1], Giovanna Giacca [1,2], Thomas Kerzel [1], Carl Mirko Mercado [1], Stefano Beretta [3], Marco Monti [3], Ivan Merelli [3], Silvia Iaia [4], Marco Genua [5], Andrea Annoni [4], Tamara Canu [6], Patrizia Cristofori [7], Sara Degl'Innocenti [7], Francesca Sanvito [7,8], Paola Maria Vittoria Rancoita [9], Renato Ostuni [2,5], Silvia Gregori [4], Luigi Naldini [2,10], Mario Leonardo Squadrito [1,2,*]

[1] Vector Engineering and In vivo Tumor Targeting Unit, San Raffaele Telethon Institute for Gene Therapy, IRCCS San Raffaele Scientific Institute, 20132 Milan, Italy

[2] Vita-Salute San Raffaele University, 20132 Milan, Italy

[3] BioInformatics Core, San Raffaele Telethon Institute for Gene Therapy, IRCCS San Raffaele Scientific Institute, 20132 Milan, Italy

[4] Mechanisms of Peripheral Tolerance Unit and Immune Core, San Raffaele Telethon Institute for Gene Therapy, IRCCS San Raffaele Scientific Institute, 20132, Milan, Italy

[5] Genomics of the Innate Immune System Unit, San Raffaele Telethon Institute for Gene Therapy, IRCCS San Raffaele Scientific Institute, 20132, Milan, Italy

[6] Preclinical Imaging Facility, IRCCS San Raffaele Scientific Institute, 20132 Milan, Italy

[7] GLP Test Facility, San Raffaele Telethon Institute for Gene Therapy, IRCCS San Raffaele Scientific Institute, 20132 Milan, Italy

[8] Pathology Unit, IRCCS San Raffaele Scientific Institute, 20132 Milan, Italy

[9] CUSSB University Center for Statistics in the Biomedical Science, Vita-Salute San Raffaele University, 20132 Milan, Italy

[10] Targeted Cancer Gene Therapy Unit, San Raffaele Telethon Institute for Gene Therapy, IRCCS San Raffaele Scientific Institute, 20132 Milan, Italy

[*] Correspondence: squadrito.mario@hsr.it (M.L.S.)

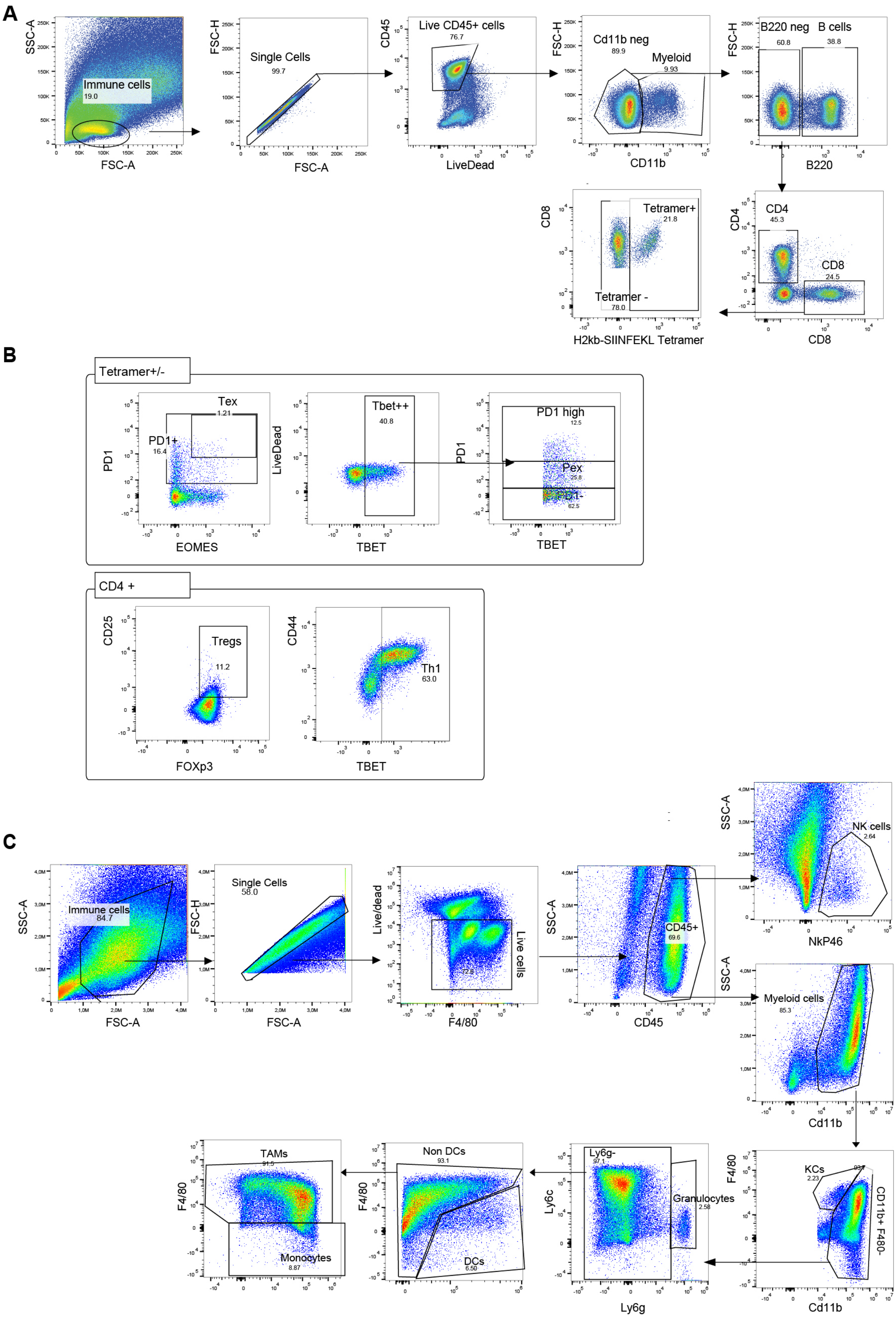

Supplementary Fig. 1. Flow cytometry gating strategy.
(A-B) Relative to figure 1D-F, J, K, O, P, figure 4C-D, G-H, O-P, figure 5C, D, J, K, figure 7G-H, K, L, Supplementary figure 2C-D, F-H, K-S, Supplementary figure 3C, I, J, Supplementary figure 7E-G, Supplementary figure 8A, C, Supplementary figure 9D, F, I, K, Supplementary figure 15C-G, I. In A, gating strategy employed to identify distinct subsets of immune cells by flow cytometry in blood, liver

and tumor samples. In B, gating strategy employed to identify distinct subsets of exhausted CD8 T cells (upper panel) or of CD4 T cells (lower panel) by flow cytometry in liver and tumor samples.
(C) Relative to supplementary figure 5F-K. Gating strategy employed to identify distinct subsets of myeloid cells by flow cytometry in liver and tumor.

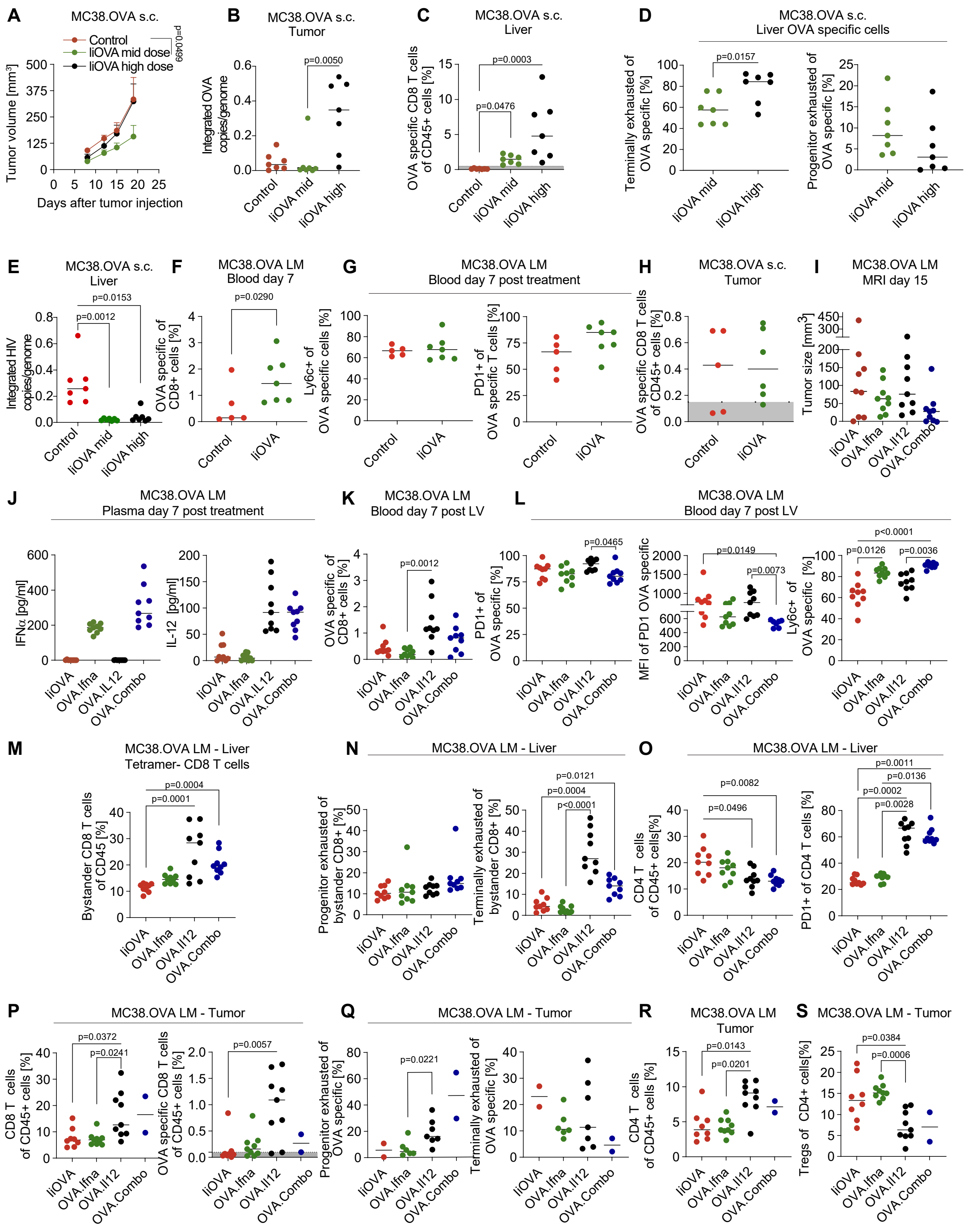

Supplementary Fig. 2. Coordinated IFNα and IL-12 expression rescues tumor reactive T cell activity.
(A-D) Delivery of liver macrophage-targeting Control LV and liOVA LV (mid or high dose) to mice before subcutaneous tumor implantation (Control LV 10^8 TU/mouse, mid dose 10^7 TU/mouse, high dose 10^8 TU/mouse). In A, s.c. tumor growth. Values represent mean + SEM. In B, LV copies per genome of the tumor by ddPCR analysis. In C-D, flow cytometry (FC) analysis of the liver. In C, values below the dark grey area are considered background noise and excluded from further analysis. n = 7 mice/group, horizontal lines represent median, in A statistical analysis by Kruskal-Wallis with Dunn's tests on area under the curve values comparing vs Control, in B and C Kruskal-Wallis with Dunn's tests, in D Mann-Whitney test. p ≤ 0.05 are shown.

(E) LV copies per genome of the liver by ddPCR analysis. (n = 7 mice/group, horizontal line represents median, statistical analysis by Kruskal-Wallis with Dunn's tests, $p \leq 0.05$ are shown).
(F-G) FC analysis of the blood (n=5,7 for Control and IiOVA, respectively; horizontal line represents median, statistical analysis by Mann-Whitney test, $p \leq 0.05$ are shown).
(H) FC analysis of the tumor (number of mice and statistic as in F).
(I) Quantification of LM volume by MRI at day 15 post tumor injection (n= 9 mice/group; horizontal line represents median, statistical analysis by Kruskal-Wallis with Dunn's tests, $p \leq 0.05$ are shown).
(J) Plasma levels of IFNα and IL-12 measured by ELISA at 7 days after treatment (n = 9 mice/group).
(K-L) FC analysis of the blood (number of mice and statistics as in I).
(M-O) FC analysis of the liver (number of mice and statistics as in I).
(P) FC analysis of the tumor (n = 8,8,9,2 mice/group for IiOVA, OVA.Ifna, OVA.Il12 and OVA.Combo treated mice, horizontal line represents median, statistical analysis by Kruskal-Wallis with Dunn's tests, $p \leq 0.05$ are shown, groups with fewer than five mice were excluded from the statistical analysis).
(Q) FC analysis of tumor OVA specific $CD8^+$ T cells (n = 2,6,7,2 mice/group for IiOVA, OVA.Ifna, OVA.Il12 and OVA.Combo treated mice, horizontal line represents median, statistical analysis by Mann-Whitney test, $p \leq 0.05$ are shown, groups with fewer than five mice were excluded from the statistical analysis).
(R-S) FC analysis of the tumor (number of mice and statistics as in P).

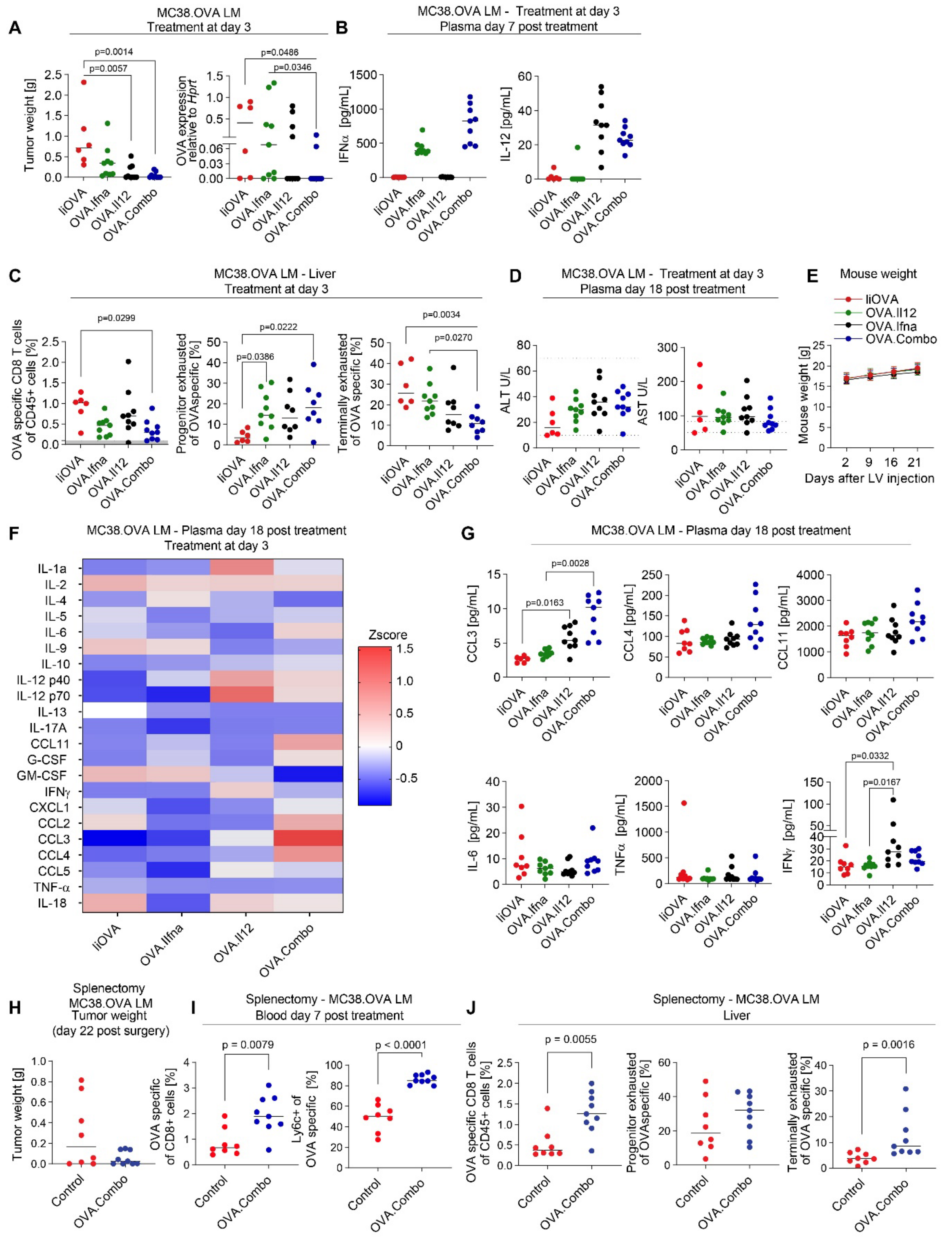

Supplementary Fig. 3. Coordinated TA, IFNα and IL-12 expression is well tolerated and activate tumor reactive T cells independently from residual expression of the transgenes in splenic macrophages.

(A-G) Treatment with liOVA, OVA.Ifna OVA.Il12 or OVA.Combo after 3 days post MC38.OVA tumor challenge (liOVA $1*10^7$ TU/mouse, OVA.Ifna total dose $1.1*10^8$ TU/mouse, OVA.Il12 total dose $1.1*10^7$ TU/mouse OVA.Combo total dose $1.2*10^8$ TU/mouse). In A, on the left, tumor weight at experiment termination, day 22, on the right OVA gene expression analysis performed on residual LM (n = 6, 9, 9, 9 mice/group, for liOVA, OVA.Ifna, OVA.Il12 and OVA.Combo treated mice, mice that completely eradicated the tumor are reported with a value of 0, horizontal line represents median, statistical

analysis by Kruskal-Wallis with Dunn's tests, $p \leq 0.05$ are shown). In B, plasma levels of IFNα and IL-12 measured by ELISA at the indicated time points after treatment (number of mice as in A). In C, FC analysis of the liver. Values below the dark grey area are considered background noise and excluded from further analysis (number of mice and statistic as in A). In D, levels of alanine aminotransferase (ALT) and aspartate aminotransferase (AST) in plasma 18 days post treatment. Dotted line represents physiological range in healthy untreated animals (number of mice and statistic as in A). In E, mouse weight monitored through the experiment (number of mice as in A, dot represent mean, lines represent SD). In F, heatmap representing the z score value for the indicated cytokines measured in the plasma of treated animals at day 18 post treatment measured by multiplexed cytokine analysis (n = 8, 9, 9, 9 mice/group, for IiOVA, OVA.Ifna, OVA.Il12 and OVA.Combo treated mice). In G, absolute level of the indicated cytokines measured by multiplexed cytokine analysis (number of mice as in F, horizontal line represents median, statistical analysis by Kruskal-Wallis with Dunn's tests, $p \leq 0.05$ are shown).
(H-K) Mice were splenectomized and, after 13 days, were challenged with MC38.OVA LM. After 7 days, mice were treated with Control or OVA.Combo (Control LV $1.2*10^8$ TU/mouse, OVA.Combo total dose $1.2*10^8$ TU/mouse). In H, tumor weight at experiment termination, day 22 after tumor placement (n = 8, 9, mice/group, for Control and OVA.Combo, horizontal line represents median). In I, FC analysis of the blood, performed at day 7 post treatment (number of mice as in H, horizontal line represents median, statistical analysis by Mann-Whitney test, $p \leq 0.05$ are shown). In J, FC analysis of the liver (number of mice and statistics ad in I)

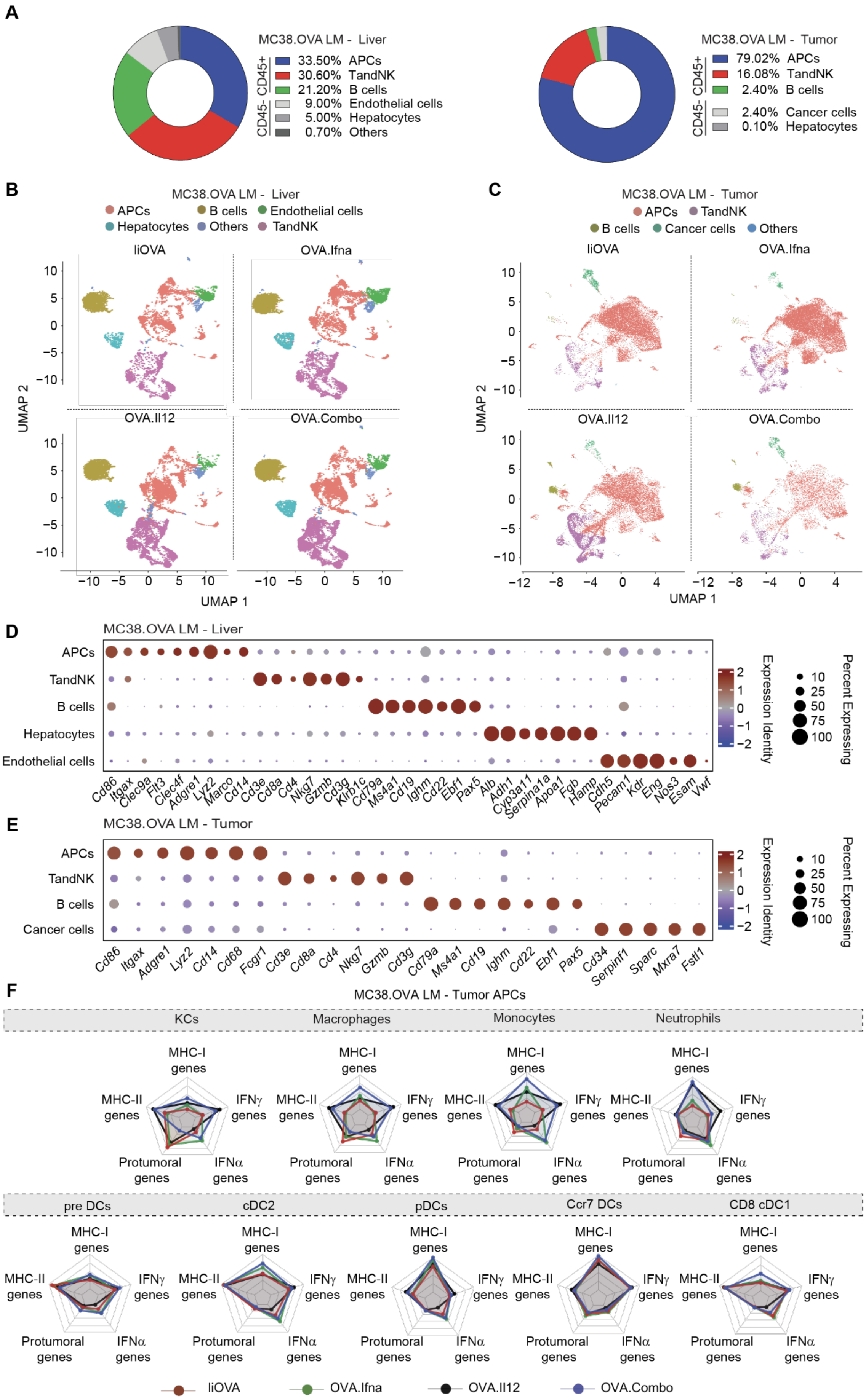
A
MC38.OVA LM - Liver
CD45+
33.50% APCs
30.60% TandNK
21.20% B cells
CD45-
9.00% Endothelial cells
5.00% Hepatocytes
0.70% Others
MC38.OVA LM - Tumor
CD45+
79.02% APCs
16.08% TandNK
2.40% B cells
CD45-
2.40% Cancer cells
0.10% Hepatocytes
B
MC38.OVA LM - Liver
APCs
B cells
Endothelial cells
Hepatocytes
Others
TandNK
liOVA
OVA.Ifna
OVA.Il12
OVA.Combo
UMAP 2
UMAP 1
C
MC38.OVA LM - Tumor
APCs
TandNK
B cells
Cancer cells
Others
liOVA
OVA.Ifna
OVA.Il12
OVA.Combo
UMAP 2
UMAP 1
D
MC38.OVA LM - Liver
APCs
TandNK
B cells
Hepatocytes
Endothelial cells
Cd86 Itgax Clec9a Flt3 Clec4f Adgre1 Lyz2 Marco Cd14 Cd3e Cd8a Cd4 Nkg7 Gzmb Cd3g Klrb1c Cd79a Ms4a1 Cd19 Ighm Cd22 Ebf1 Pax5 Alb Adh1 Cyp3a11 Serpina1a Apoa1 Fgb Hamp Cdh5 Pecam1 Kdr Eng Nos3 Esam Vwf
Expression Identity
Percent Expressing
E
MC38.OVA LM - Tumor
APCs
TandNK
B cells
Cancer cells
Cd86 Itgax Adgre1 Lyz2 Cd14 Cd68 Fcgr1 Cd3e Cd8a Cd4 Nkg7 Gzmb Cd3g Cd79a Ms4a1 Cd19 Ighm Cd22 Ebf1 Pax5 Cd34 Serpinf1 Sparc Mxra7 Fstl1
Expression Identity
Percent Expressing
F
MC38.OVA LM - Tumor APCs
KCs
Macrophages
Monocytes
Neutrophils
pre DCs
cDC2
pDCs
Ccr7 DCs
CD8 cDC1
MHC-I genes
MHC-II genes
IFNγ genes
IFNα genes
Protumoral genes
liOVA
OVA.Ifna
OVA.Il12
OVA.Combo

Supplementary fig. 4. Concurrent IFNα and IL-12 expression increases MHC-I and MHC-II presentation in liver and liver metastases.
(A) Pie chart indicating the fraction of distinct subset of $CD45^+$ and $CD45^-$ cells in our single cell dataset, obtained from sorting $CD45^+$ cells from the liver (left panel) or the matched liver metastases (right panel). N = 2, 3, 3, 3 mice/group for IiOVA, OVA.Ifna, OVA.Il12 and OVA.Combo.
(B-C) UMAP projection of scRNA-seq of the whole dataset for the indicated group and tissue. Number of mice as in A.
(D-E) Fraction of positive cells and scaled averaged expression of top markers and know genes expressed in the annotated cell types in liver (D) and matched liver metastases (E). Number of mice as in A.
(F) Combined gene expression score for genes belonging to the indicated categories in the distinct tumor myeloid populations. Number of mice as in A.

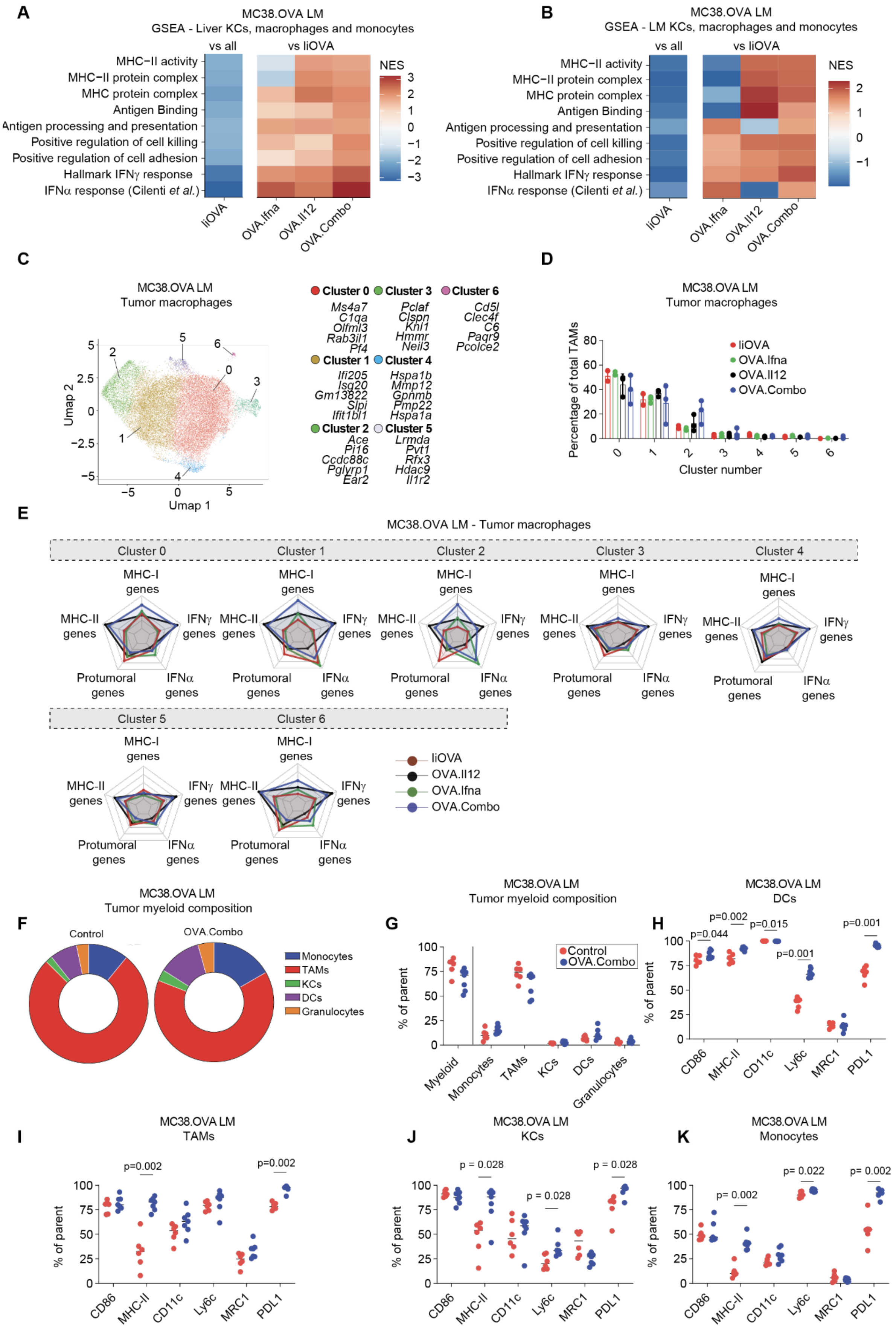
A
MC38.OVA LM
GSEA - Liver KCs, macrophages and monocytes
vs all
vs liOVA
NES
MHC−II activity
MHC−II protein complex
MHC protein complex
Antigen Binding
Antigen processing and presentation
Positive regulation of cell killing
Positive regulation of cell adhesion
Hallmark IFNγ response
IFNα response (Cilenti et al.)
liOVA
OVA.Ifna
OVA.Il12
OVA.Combo
B
MC38.OVA LM
GSEA - LM KCs, macrophages and monocytes
C
MC38.OVA LM
Tumor macrophages
Umap 1
Umap 2
Cluster 0
Ms4a7
C1qa
Olfml3
Rab3il1
Pf4
Cluster 1
Ifi205
Isg20
Gm13822
Slpi
Ifit1bl1
Cluster 2
Ace
Pi16
Ccdc88c
Pglyrp1
Ear2
Cluster 3
Pclaf
Clspn
Knl1
Hmmr
Neil3
Cluster 4
Hspa1b
Mmp12
Gpnmb
Pmp22
Hspa1a
Cluster 5
Lrmda
Pvt1
Rfx3
Hdac9
Il1r2
Cluster 6
Cd5l
Clec4f
C6
Paqr9
Pcolce2
D
MC38.OVA LM
Tumor macrophages
Percentage of total TAMs
Cluster number
liOVA
OVA.Ifna
OVA.Il12
OVA.Combo
E
MC38.OVA LM - Tumor macrophages
Cluster 0
Cluster 1
Cluster 2
Cluster 3
Cluster 4
Cluster 5
Cluster 6
MHC-I genes
IFNγ genes
IFNα genes
Protumoral genes
MHC-II genes
liOVA
OVA.Il12
OVA.Ifna
OVA.Combo
F
MC38.OVA LM
Tumor myeloid composition
Control
OVA.Combo
Monocytes
TAMs
KCs
DCs
Granulocytes
G
MC38.OVA LM
Tumor myeloid composition
% of parent
Control
OVA.Combo
Myeloid
Monocytes
TAMs
KCs
DCs
Granulocytes
H
MC38.OVA LM
DCs
p=0.044
p=0.002
p=0.015
p=0.001
p=0.001
CD86
MHC-II
CD11c
Ly6c
MRC1
PDL1
I
MC38.OVA LM
TAMs
p=0.002
p=0.002
J
MC38.OVA LM
KCs
p = 0.028
p = 0.028
p = 0.028
K
MC38.OVA LM
Monocytes
p= 0.002
p= 0.022
p= 0.002

Supplementary fig. 5. Concurrent delivery of IFNα and IL-12 reprograms tumor myeloid cells
(A-B) GSEA of scRNA-seq data showing NES for selected GO terms calculated based on genes differentially expressed in KCs, macrophages and monocytes in the indicated comparisons (n = 2, 3, 3, 3 mice/group for IiOVA, OVA.Ifna OVA.Il12 and OVA.Combo).
(C) UMAP projection of scRNA-seq of tumor macrophage subcluster.
(D) Percentage of TAM sublcuster in the distinct treatment cohorts. Horizontal line represents mean, error bars represent the SEM. Number of mice as in A.
(E) Combined gene expression score for genes belonging to the indicated categories in the distinct TAMs clusters. Number of mice as in A.
(F-K) Mice were injected intrahepatically with MC38.OVA cells and after 7 days treated with OVA.Combo (total dose $1.2*10^8$ TU/mouse) or left untreated. In F-G, fraction of distinct subset of myeloid cells infiltrating the liver metastases. In H-K flow cytometry analysis of the tumor (n = 6,7, Control UT and OVA.Combo respectively, horizontal line represents median, statistical analysis by multiple Mann-Whitney test, $p \leq 0.05$ are shown).

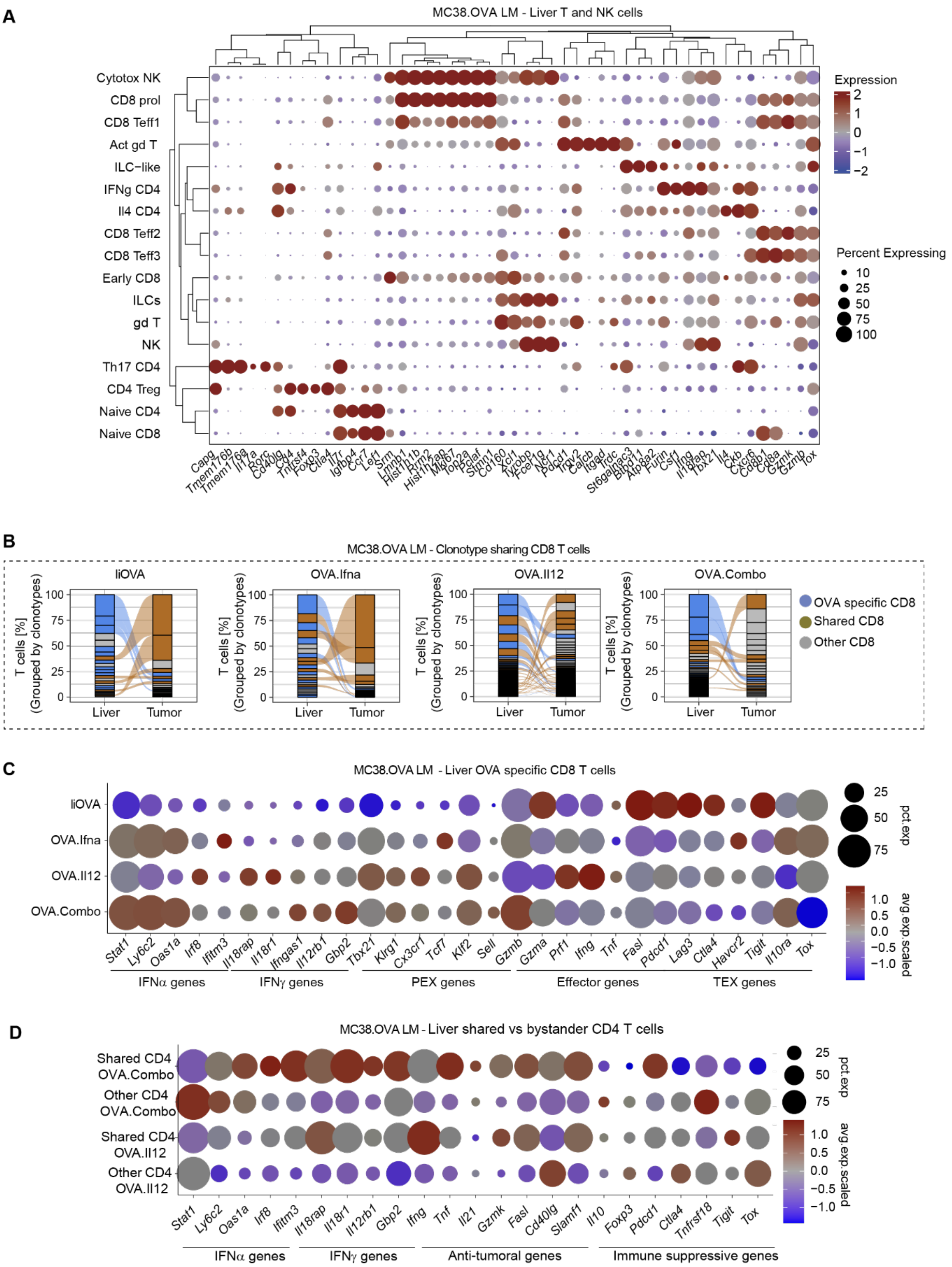

Supplementary fig. 6. Concurrent IFNα and IL-12 expression ameliorates the fitness of CD4^{+} and TA specific CD8^{+} T cells.

(A) Dotplot showing the average and percent of expression of top genes for each cluster of liver T and NK cells. (n = 2, 3, 3, 3 mice/group for liOVA, OVA.Ifna OVA.Il12 and OVA.Combo).

(B) Clonotype sharing of OVA specific and bystander CD8^{+} T cells between liver and tumor, grouped by TCR clonotype. Number of mice as in A.

(C) Expression of selected genes belonging to the indicated categories in liver OVA specific CD8^{+} T cells. Number of mice as in A.

(D) Expression of selected genes belonging to the indicated categories in liver shared vs non shared $CD4^+$ T cells in OVA.Combo or OVA.Il12 treated animals (number of mice as in A).

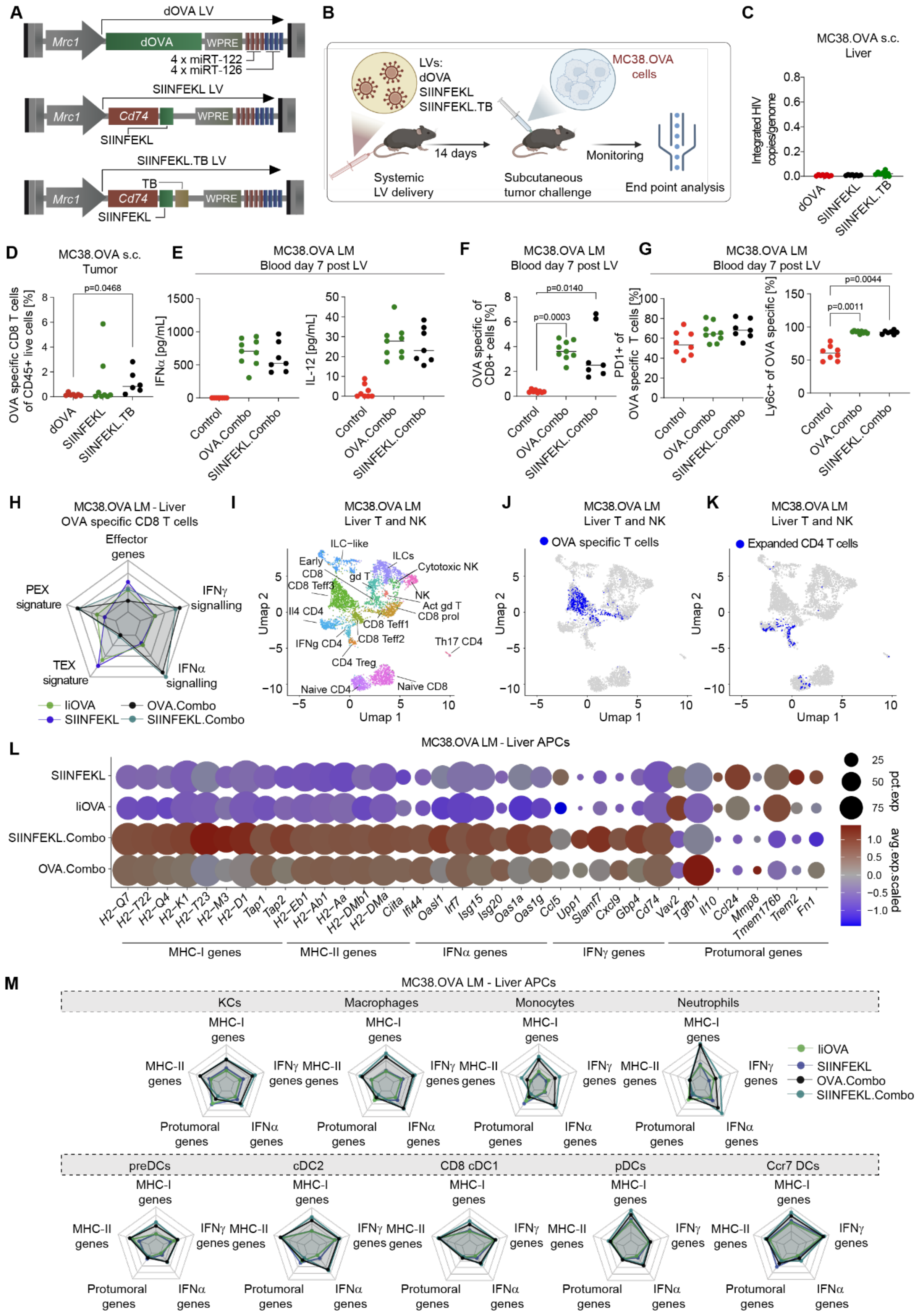

Supplementary fig. 7. Coordinated delivery of IFNα and IL-12 activates TA specific CD8⁺ T cells independently of the delivery of MHC-II loadable peptides.
(A) Schematics of the distinct LVs employed.

(B) Schematic of the therapeutic experiment in panels A-D of Figure 4 and C-D of supplementary fig. 7. Illustration from BioRender.
(C) LV copies per genome of the liver by ddPCR analysis (n = 7, 8, 6 mice/group for dOVA, SIINFEKL or SIINFEKL.TB treated mice, horizontal line represents median, statistical analysis by Kruskal-Wallis with Dunn's tests, $p \leq 0.05$ are shown).
(D) FC analysis of the tumor (number of mice and statistics as in C).
(E) Plasma levels of IFNα and IL-12 measured by ELISA at 7 days after treatment (n = 8,9,7,7,19 mice/group for Control, OVA.Combo, dOVA.Combo, SIINFEKL.Combo or SIINFEKL.TB.Combo respectively, horizontal line represents median).
(F-G) FC analysis of the blood, performed at day 14 post tumor injection (number of mice and statistics as in E, statistical analysis by Kruskal-Wallis with Dunn's tests, $p \leq 0.05$ are shown).
(H) Combined gene expression score for genes belonging to the indicated categories in liver OVA specific $CD8^+$ T cells (n= 1 mouse/group).
(I) UMAP projection of scRNA-seq of liver T and NK cell subclusters.
(J) UMAP projection of scRNA-seq indicating OVA specific $CD8^+$ T cells.
(K) UMAP projection of scRNA-seq indicating expanded $CD4^+$ T cells.
(L) Expression of selected genes belonging to the indicated categories in liver APCs (n= 1 mouse/group).
(M) Combined gene expression score for genes belonging to the indicated categories in the indicated groups (n= 1 mouse/group).

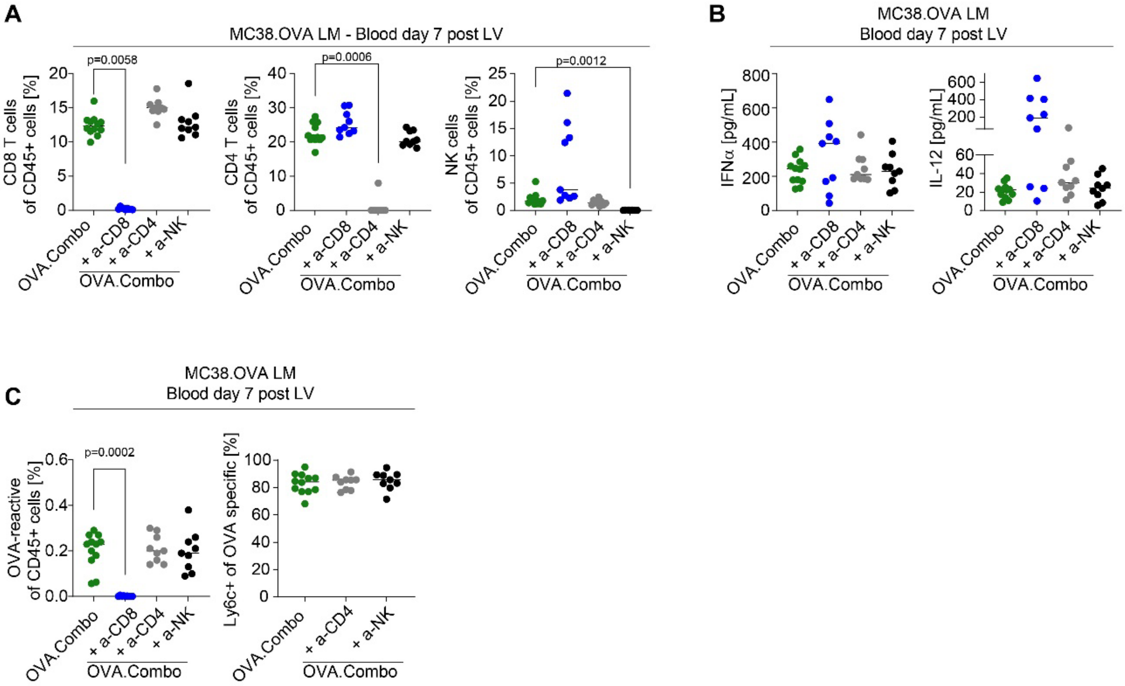

Supplementary fig. 8. Coordinated delivery of IFNα and IL-12 activates TA specific CD8$^+$ T cells independently of CD4$^+$ T cell help.

(A-C) Depletion of CD4$^+$, CD8$^+$ or NK cells. Mice were injected intrahepatically with MC38.OVA cells and after 6 days, injected IP with 0.2 mg of a-CD4, a-CD8 or a-NK. On day 7 mice were injected with OVA.Combo (total dose 1.2*10^8 TU/mouse) and then with the respective mabs IP twice weekly until experiment termination. In A, FC analysis of the blood 7 days post LV injection (n = 12,9,9,9 mice/group for OVA.Combo, OVA.Combo + a-CD4, OVA.Combo + a-CD8, OVA.Combo + a-NK respectively, horizontal line represents median, statistical analysis by Kruskal-Wallis with Dunn's tests comparing all groups vs OVA.Combo, $p \leq 0.05$ are shown). In B, plasma levels of IFNα and IL-12 measured by ELISA at 7 days after treatment (number of mice as in A). In C, FC analysis of the blood (number of mice and statistics as in A).

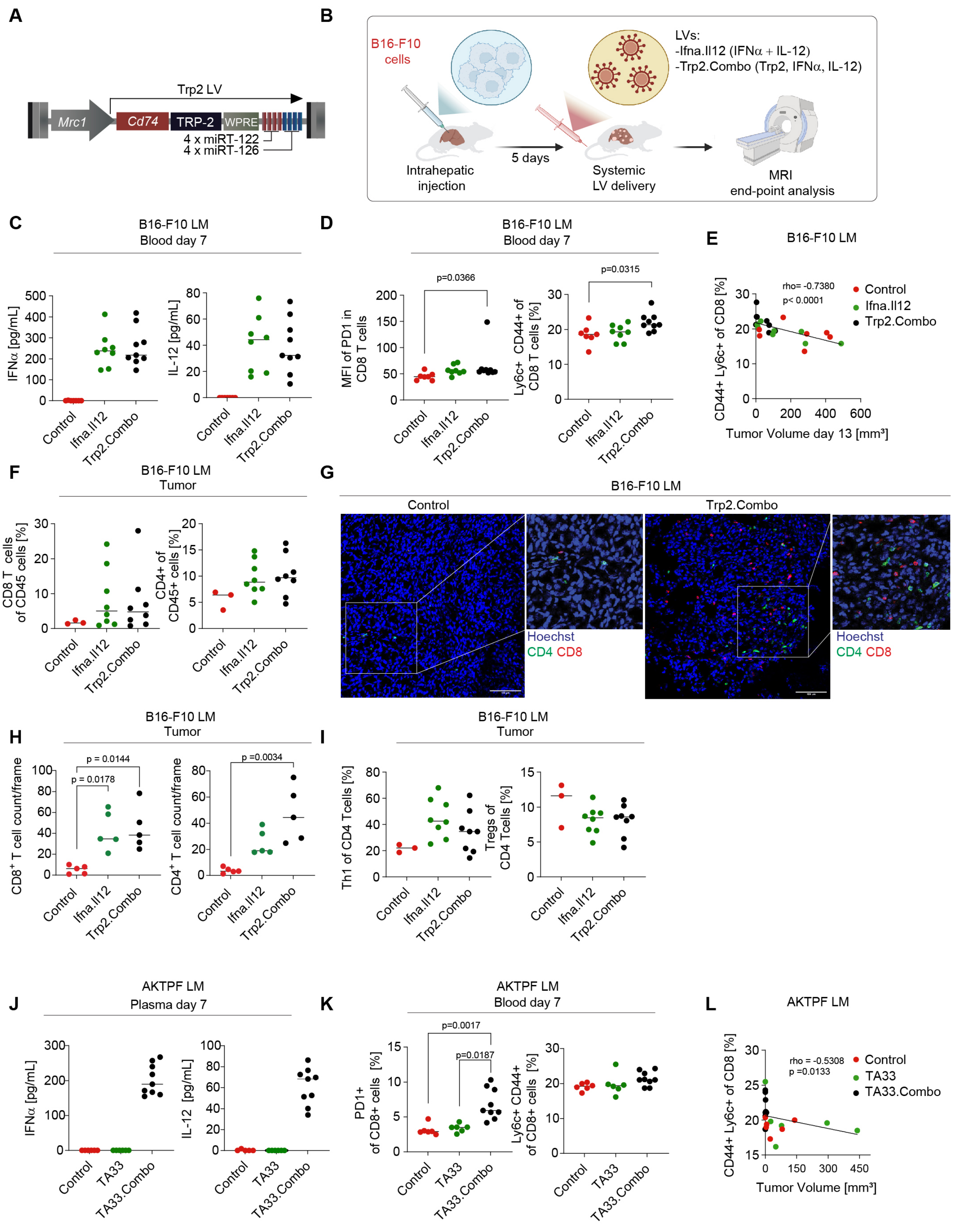

Supplementary fig. 9. Concurrent delivery of naturally occurring TAs together with IFNα and IL-12 inhibits melanoma and CRC LM growth by expanding and reprogramming TA reactive CD8^{+} T cells.

(A) Schematics of the Trp2 LV.

(B) Schematic of the experiments shown in panels A-E of Figure 5 and C-G of Supplementary fig.9. Illustration from BioRender.

(C) Plasma levels of IFNα and IL-12 measured by ELISA at 7 days post treatment (n= 7, 8, 9 mice/group, for Control untreated, Ifna.Il12 or Trp2.Combo treated mice; horizontal line represents median).

(D) FC analysis of the blood, performed at day 14 post tumor injection (number of mice as in C, horizontal line represents median, statistical analysis by Kruskal-Wallis with Dunn's tests, $p \leq 0.05$ are shown).
(E) Correlation between circulating Ly6c+ CD44+ CD8 T cells and tumor volume measured by MRI at day 13 (n of mice as in C, statistical analysis by Spearman correlation).
(F) FC analysis of the tumor (n= 3, 8,8 mice/group for Control untreated, Ifna.Il12 and Trp2.Combo treated animals).
(G) Representative immunofluorescence images obtained by confocal microscopy from the tumor of mice untreated or treated with Trp2.Combo; Hoechst staining for nuclei (blue), CD8 (red) and Trp2 (green). Scale bar 100 μm.
(H) Quantification of $CD8^+$ and $CD4^+$ T cells infiltrating in the tumor by immunofluorescence imaging (n= 5 mice/group, horizontal line represents median statistical analysis by Kruskal-Wallis with Dunn's tests, $p \leq 0.05$ are shown).
(I) FC analysis of the tumor (number of animals as in F).
(J) Plasma levels of IFNα and IL-12 measured by ELISA at 7 days post treatment (n= 6, 6, 9 mice/group, for Control untreated, TA33 or TA33.Combo treated mice; horizontal line represents median).
(K) FC analysis of the blood, performed at day 14 post tumor injection (number of mice as in J, horizontal line represents median, statistical analysis by Kruskal-Wallis with Dunn's tests, $p \leq 0.05$ are shown).
(L) Correlation between circulating Ly6c+ CD44+ CD8 T cells and tumor volume measured by MRI at day 27 (n of mice as in J, statistical analysis by Spearman correlation).

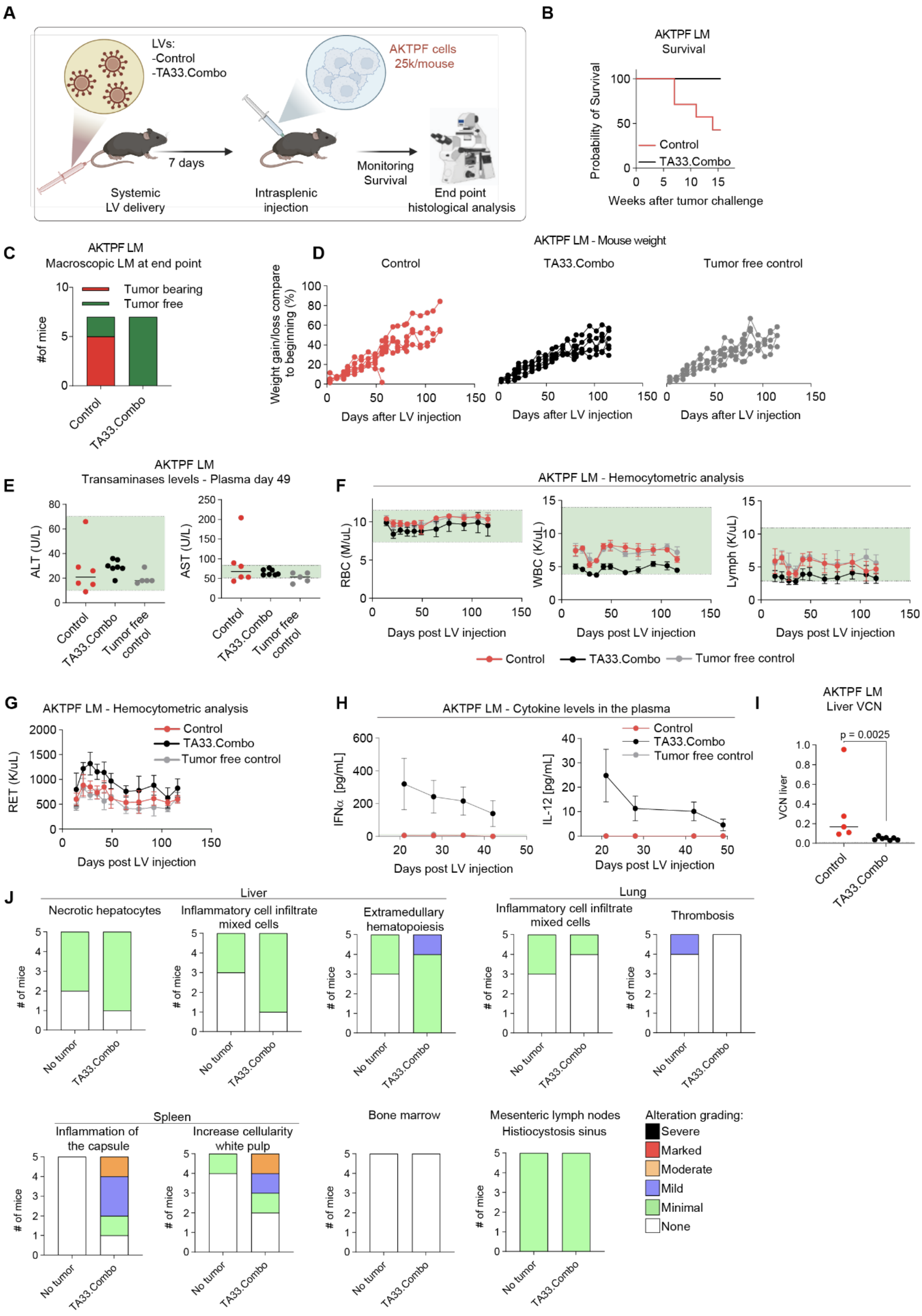

Supplementary fig. 10. Concurrent delivery of naturally occurring TAs together with IFNα and IL-12 prevents tumor engraftment and is well tolerated.

(A-J) Delivery of Control LV or TA33.Combo LV to mice before tumor challenge (Control LV 1.2x10^8 TU/mouse, TA33.Combo 1.2 x10^8 total TU/mouse). In A, Schematic of the experiment shown in panels A-J. Illustration from BioRender. In B, probability of survival (n= 6, 7 for Control and TA33.Combo treated

mice respectively). In C, contingency table indicating tumor free and tumor bearing mice at experiment termination (day 115 after LV treatment). In D, mouse weight monitored through the experiment (number of mice as in B plus n = 5 tumor free mice). In E, ALT and AST levels measured in the plasma at day 49 post treatment (n of mice as in D, statistical analysis by Kruskal-Wallis with Dunn's tests, $p \leq 0.05$ are shown). In F, counts of red blood cell (RBC), white blood cell (WBC) lymphoid cells (Lymph) measured by hemocytometric analysis (n of mice as in D). In G, counts of reticulocytes (RET) measured by hemocytometric analysis (n of mice as in D). In H, levels of INFα and IL-12 measured by ELISA at different time points (n of mice as in D). In I, liver vector copy number analysis (n= 5, 7 for Control and TA33.Combo treated mice respectively, statistical analysis by Mann-Whitney test, $p \leq 0.05$ are shown). In J, histopathologic analysis of the indicated organs at day 115 upon LV injection (n=5 mice/group).

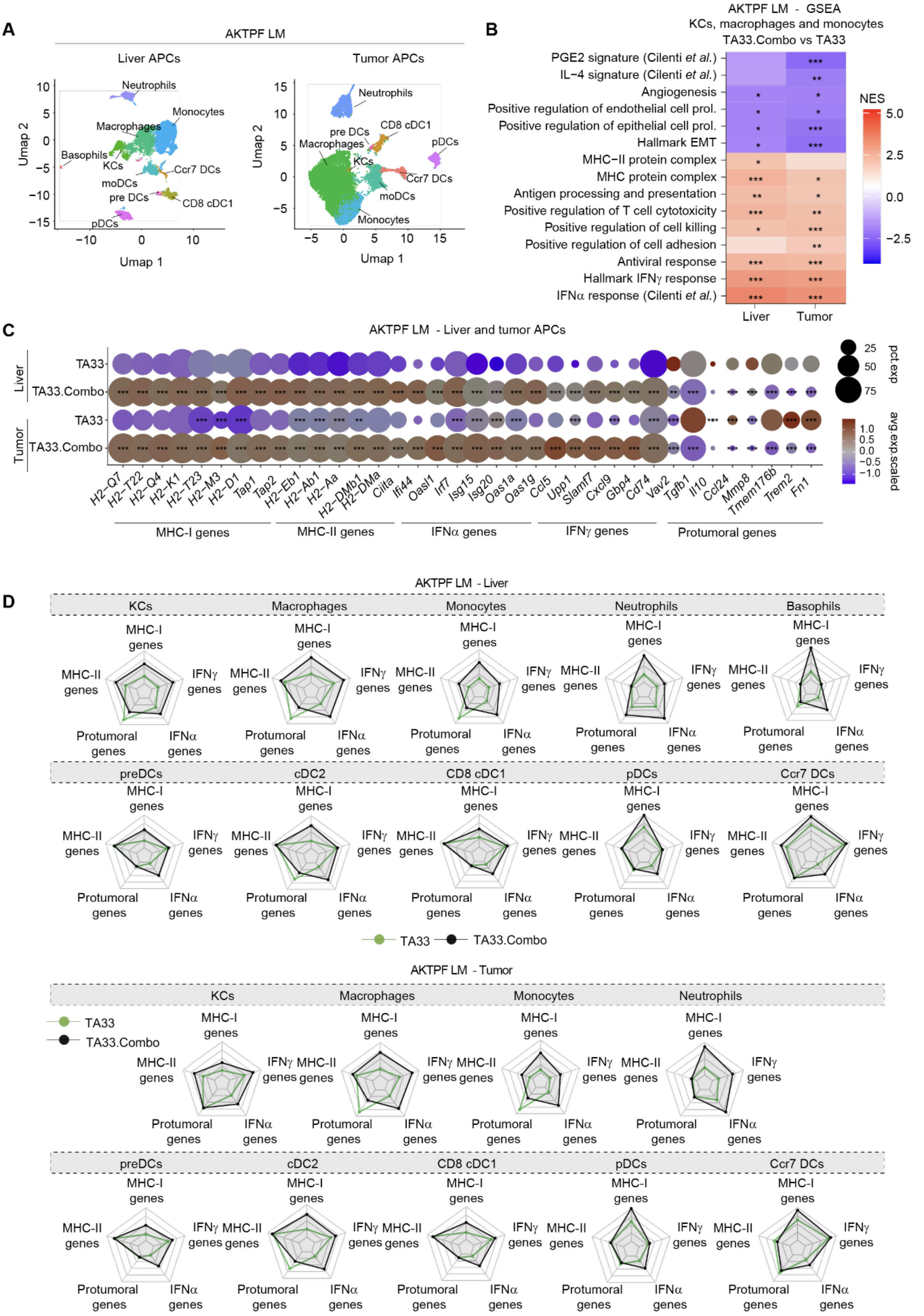

Supplementary fig. 11. IFNα and IL-12 reprograms myeloid cells in the LM microenvironment.
(A) UMAP projection of scRNA-seq of APCs subcluster from the indicated tissue.
(B) GSEA of scRNA-seq data showing NES for selected GO terms calculated based on genes differentially expressed in the indicated tissue, cell type and comparisons (n = 3 mice/group; statistical

analysis by an adaptive multi-level split Monte-Carlo scheme; *: padj <0.05; **: padj <0.005; ***: padj <0.0005).

(C) Expression of selected genes belonging to the indicated categories in APCs from the indicated tissue (n= 3 mouse/goup; statistical analysis by Wilcoxon test with Bonferroni correction, compared with liver TA33. *: padj <0.05; **: padj <0.01; ***: padj <0.001).

(D) Combined gene expression score for genes belonging to the indicated categories in the different cell populations from the indicated tissue and cohort. Number of mice as in B.

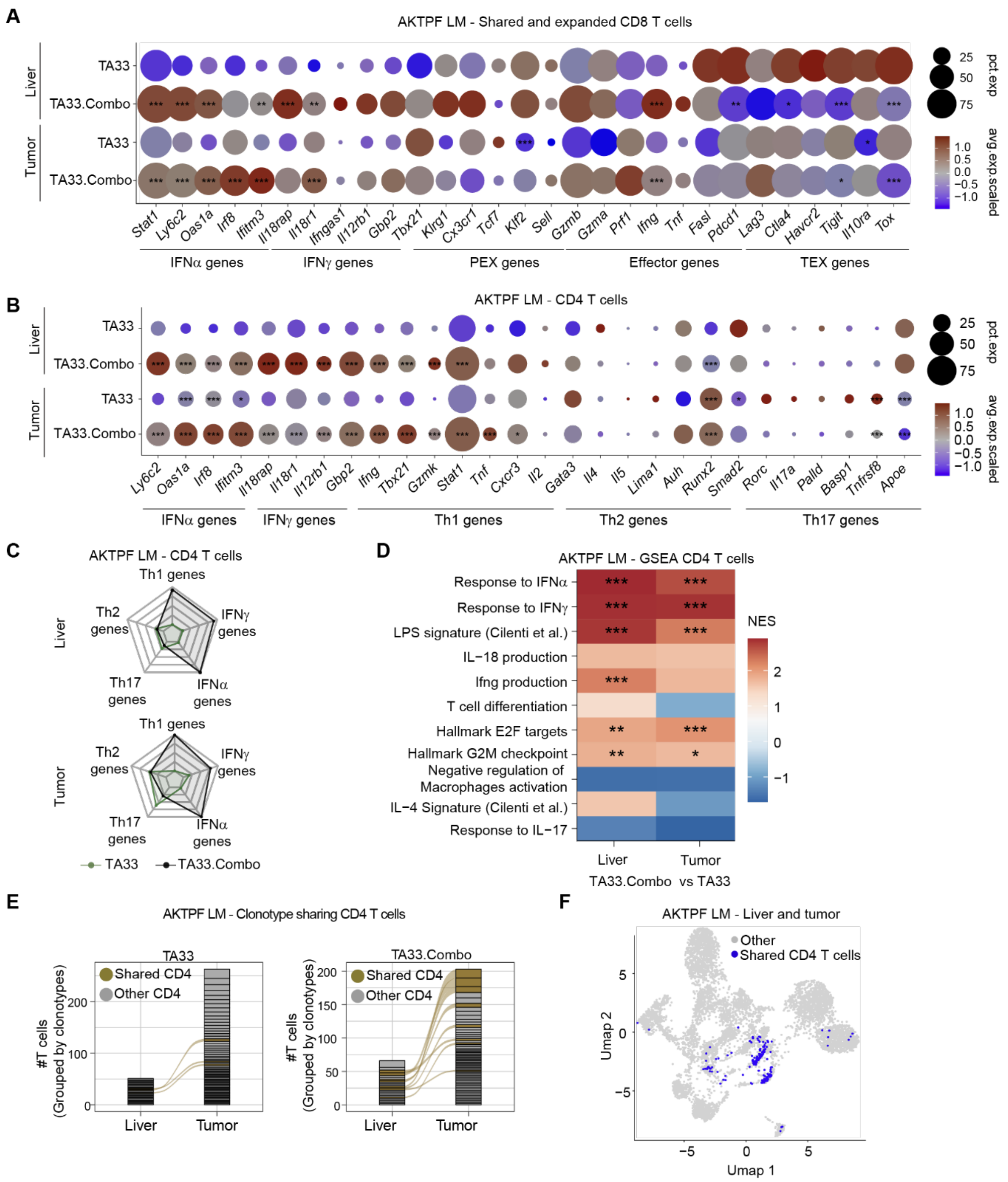

Supplementary fig. 12. IFNα and IL-12 reprograms CD4 and CD8 T cells in the LM microenvironment.

(A) Expression of selected genes belonging to the indicated categories in putative tumor reactive CD8^{+} T cells from the indicated tissue (n = 3 mice/group; statistical analysis by Wilcoxon test with Bonferroni correction, compared with the liver TA33. *: padj <0.05; **: padj <0.005; ***: padj <0.0005).

(B) Expression of selected genes belonging to the indicated categories in CD4^{+} T cells from the indicated tissue (number of mice and statistic as in A).

(C) Combined gene expression score for genes belonging to the indicated categories in the indicated tissue in CD4^{+} T cells (number of mice as in A).

(D) GSEA of scRNA-seq data showing NES for selected GO terms calculated based on genes differentially expressed in the indicated tissue, cell type and comparisons (n = 3 mice/group; statistical analysis by an adaptive multi-level split Monte-Carlo scheme; *: padj <0.05; **: padj <0.005; ***: padj <0.0005).

(E) Clonotype sharing between liver CD4^{+} T cells, grouped by TCR clonotype (n= 3 mouse/group).

(F) UMAP projection of scRNA-seq indicating shared CD4^{+} T cells.

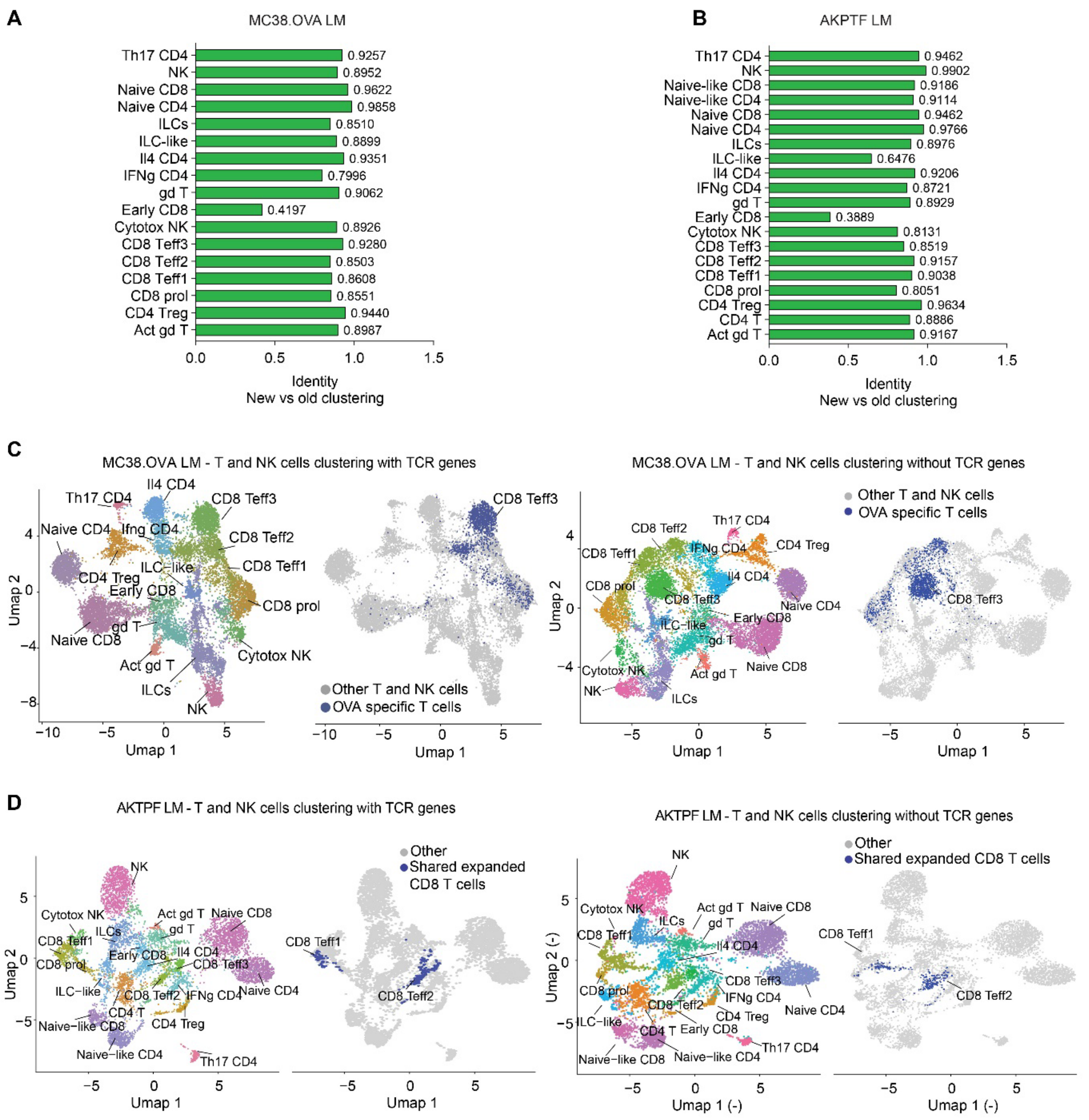

Supplementary fig. 13. Removal of TCR genes does not affect cluster and cell type identification. (A,B) Percentage of identity between cells identified with or without the removal of TCR genes.
(C) UMAP projection of scRNA-seq of liver T and NK cells clustered with (left, as in figure 3 A;E) or without TCR genes, showing enrichment of OVA-specific CD8 T cells in the CD8 Teff cluster. Data derived from CD45+ cells sorted from MC38.OVA LM.
(D) UMAP projection of scRNA-seq of liver T and NK cells clustered with (left, as in figure 6B) or without TCR genes, showing enrichment of putative tumor reactive CD8 T cells in the CD8 Teff clusters. Data derived from CD45+ cells sorted from AKTPF LM.

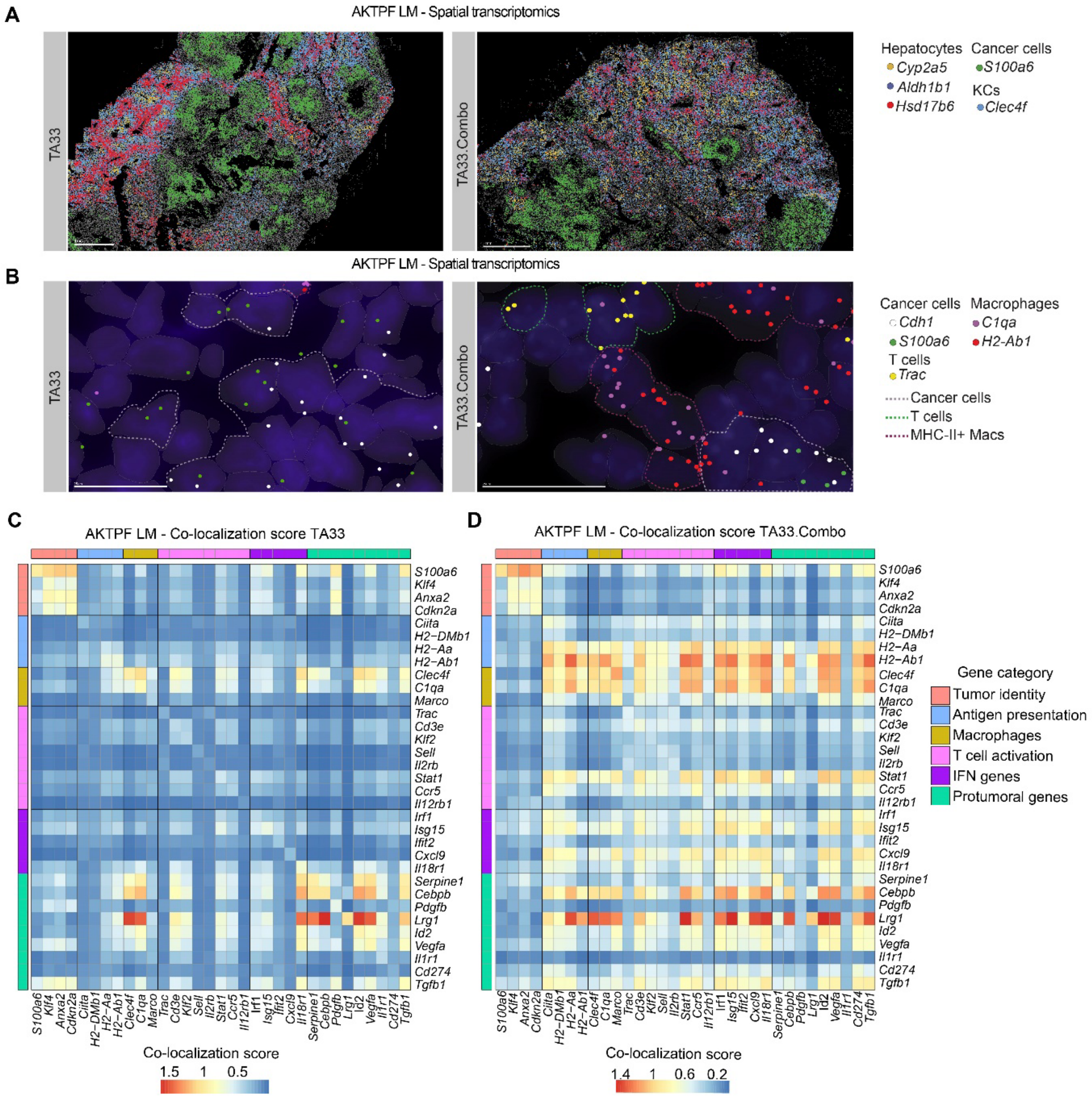

Supplementary fig. 14. IFNα and IL-12 rewire immune circuits within the metastatic microenvironment enabling TA specific T cell activity.

(A) Representative images showing the spatial distribution of selected transcripts on liver tissue section, collected from mice treated with TA33 (left) or TA33.Combo (right) LV, as described in Methods. Selected samples included both healthy liver parenchyma and liver metastases. The distribution of the selected transcripts identified hepatocytes (enriched in *Cyp2a5*, *Aldh1b1*, and *Hsd17b6*), cancer cells (*S100a6*) and KCs (*Clec4f*).

(B) Representative images showing the spatial distribution of transcripts associated to cancer cells (*Cdh1*, *S100a6*), T cells (*Trac*) and macrophages (*C1qa*, *H2-Ab1*). Enlargements of the liver sections in (A) highlights the proximity of the three cell types indicated by dashed lines.

(C,D) Heatmaps showing the co-localization score of the indicated transcripts detected on the liver tissue sections analyzed in (A).

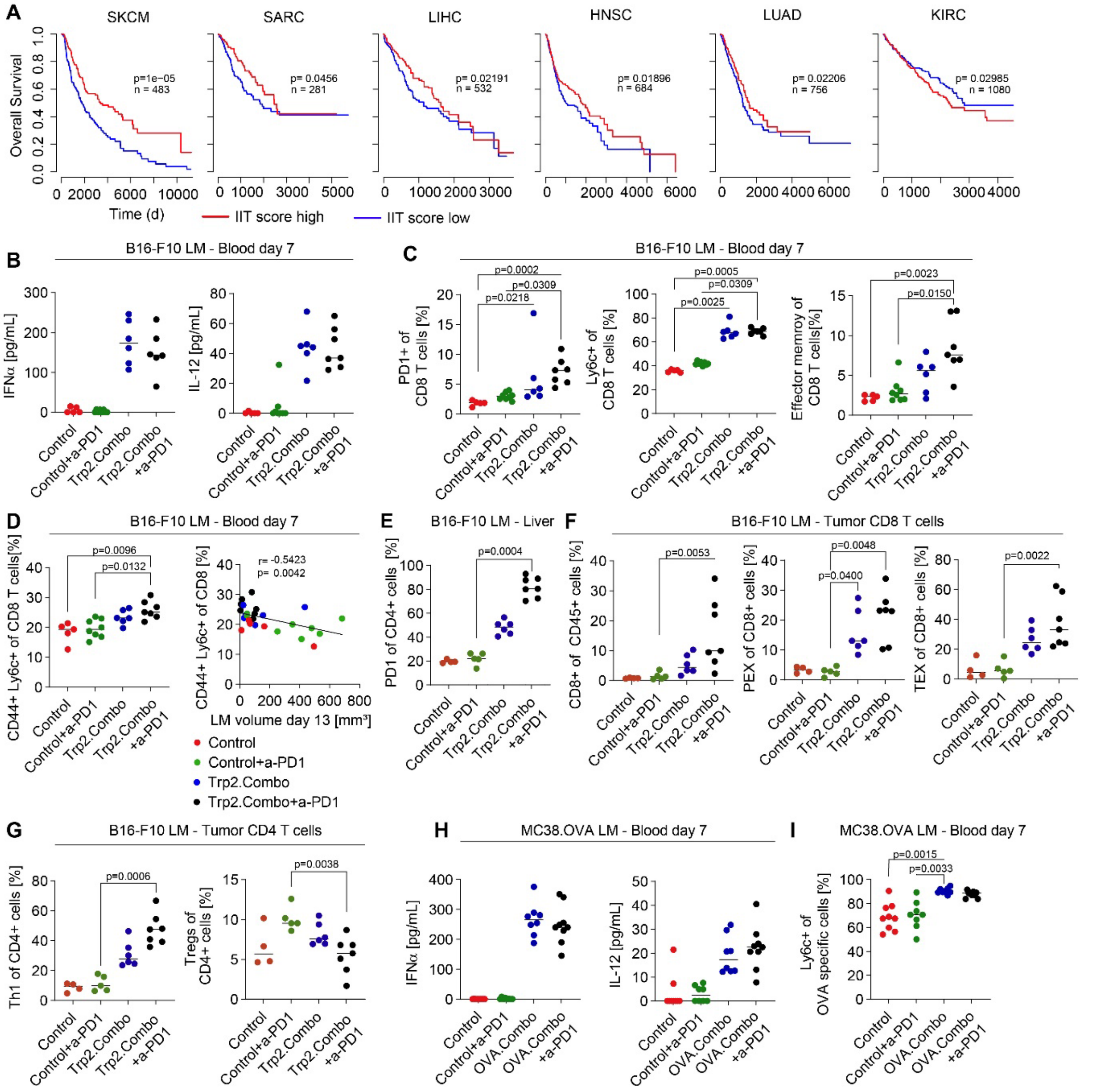

Supplementary fig. 15. TA, IFNα and IL-12 LVs restore response to immunotherapy by expanding TA-specific PEX CD8⁺ T cells.

(A) Kaplan–Meier survival curves of patients with high vs low IIT score. Skin Cutaneous Melanoma (SKCM, n=483), Sarcoma (SARC, n=281), Liver Hepatocellular Carcinoma (LIHC, n=532), Head and neck squamous cell carcinoma (HNSC, n=684) Lung Adenocarcinoma (LUAD, n=756), Kidney Renal Clear Cell Carcinoma (KIRC, n=1080). The survival distributions were compared using a Cox proportional hazards model, with the p value derived from the Wald test of the Cox regression.

(B) Plasma levels of IFNα and IL-12 measured by ELISA at 7 days post treatment (n= 5, 8, 6, 7 mice/group, for Control untreated, Control + a-PD1, Trp2.Combo or Trp2.Combo + a-PD1 treated mice; horizontal line represents median)

(C) FC analysis of the blood (number of mice as in B, horizontal line represents median, statistical analysis by Kruskal-Wallis with Dunn's tests, $p \leq 0.05$ are shown).

(D) On the left, FC analysis of the blood (number of mice as in B), on the right correlation between circulating Ly6c+ CD44+ $CD8^+$ T cells and tumor volume measured by MRI at day 13 (n of mice as in B, statistical analysis by Spearman correlation).

(E) FC analysis of the liver (n= 3, 5, 6, 7 mice/group, for Control untreated, Control + a-PD1, Trp2.Combo or Trp2.Combo + a-PD1 treated mice; horizontal line represents median, statistical analysis by Kruskal-Wallis with Dunn's tests, $p \leq 0.05$ are shown, groups with fewer than five mice were excluded from the statistical analysis).

(F-G) FC analysis of the tumor (n= 4, 5, 6, 7 mice/group, for Control untreated, Control + a-PD1, Trp2.Combo or Trp2.Combo + a-PD1 treated mice; horizontal line represents median, statistical

analysis by Kruskal-Wallis with Dunn's tests, p ≤ 0.05 are shown, groups with fewer than five mice were excluded from the statistical analysis).
(H) Plasma levels of IFNα and IL-12 measured by ELISA at 7 days post treatment (n= 9, 7, 8, 9 mice/group, for Control untreated, Control + a-PD1, OVA.Combo or OVA.Combo + a-PD1 treated mice; horizontal line represents median)
(I) FC analysis of the blood (number of mice as in H; horizontal line represents median, statistical analysis by Kruskal-Wallis with Dunn's tests, p ≤ 0.05 are shown).

Supplementary Tables

Primer/probe name	Sequence (5' to 3')
HIV primer Fw	TACTGACGCTCTCGACC
HIV primer Rev	TCTCGACGCAGGACTCG
HIV probe (FAM)	ATCTCTCTCCTTCTAGCCTC
Sema3a primer Fw	ACCGATTCCAGATGATTGGC
Sema3a primer Rev	TCCATATTAATGCAGTGCTTG
Sema3a probe (HEX)	AGAGGCCTGTCCTGCAGCTCATGG
TRP2.FWD	AAAAAAACGCGTTTTCCCCGAGTCTGCATGA
TRP2.REV	AAAAAAGTCGACTCAGCTAGCGAGAGTTGTGGACCAAAC
A85B_S	CTAGCTTCCAGGACGCCTACAATGCTGCTGGCGGCCACAACGCTGTGTTCTGAG
A85b_AS	TCGACTCAGAACACAGCGTTGTGGCCGCCAGCAGCATTGTAGGCGTCCTGGAAG
Ii.OVA.s	AAAAAGGATCCACCATGGATGACCAACGC
OVA.SIINFEKL	AAAAGTCGACTAGCTAGCAACGTTAGAACTGGTCCAT

Supplementary Table 1. List of non-commercial primers and probes employed in this study

Target	TaqMan assay ID
Hprt	Mm03024075_m1
OVAL	Gg03366807_m1

Supplementary Table 2. List of commercial TaqMan assays employed in this study

Gene name	nmer
Chd4	ATVECAQL
Emp3	VSGIVYIHL
Trp2	SVYDFFVWL

Supplementary Table 3. Peptides employed in this study

n of peptide	Gene name	nmer
1	*Abcb10*	LSIPYGSV

2	*B3gnt3*	WSKYFIPTL
3	*Babam1*	QTHSSYSLL
4	*Bcam*	SAPEELFVFL
5	*Cdt1 (1)*	TVYPMSYRF
6	*Cdt1 (2)*	MSYRFRQE
7	*Cdt1 (3)*	VEMFHSMDTI
8	*Cdh1*	TQEVFEGSV
9	*Cox11*	RTVVYAEL
10	*Rnaseh1*	AAVVSKDTF
11	*Gale*	KSLVFSSSA
12	*Gpi1*	INYTEDRAV
13	*Greb1*	CSSSLFTPL
14	*Heatr5a*	KSLVFAALEL
15	*Hmgcs1*	IGVFSYGSGL
16	*Lars2*	VMAMSMLTL
17	*Nomo1*	VVLLDSTL
18	*Nup62*	VSFGLGSSTL
19	*Romo1*	TFGTFTAI
20	*Scpep1*	VQLWLLLL
21	*Serinc3*	YNYSFFHL
22	*Stt3b*	LSAVAFSNV
23	*Sugp1*	STGSFPAL
24	*Syne2*	VLHHFALSV
25	*Tm7sf3*	VGFIFLGFF
26	*Tm7sf3*	KVFSTLFALL
27	*Ufd1*	VTYSKFQPQ
28	*Xylt2*	TAYTAFARLG
29	*Ggh*	LIYKVYPI
30	*Gsap*	NAIAFLNL

Supplementary Table 4. List of peptides pooled, relative to figure 5 L and M.

	pool 1	pool 2	pool 3	pool 4	pool 5
pool 7	1	7	13	19	25
pool 8	2	8	14	20	26
pool 9	3	9	15	21	27
pool 10	4	10	16	22	28
pool 11	5	11	17	23	29
pool 12	6	12	18	24	30

Supplementary Table 5. Pooling strategy, relative to figure 5 L and M.

**Target**	**Clone**	**Conjugation**	**Producer**	**Working dilution**
CD11b	M1/70	BV710	BioLegend	1/100
CD279 (PD1)	29F.1A12	PE-Cy7	BioLegend	1/100
CD4	RM4-5	BUV737	BD Horizon	1/100
CD44	IM7	BV605	BD Horizon	1/100
CD45	30-F11	BV510	BioLegend	1/200
CD62L	MEL-14	BV786	eBioscience	1/100
CD8	53-6.7	FITC	BD Pharmagen	1/200
Ly6c	HK1.4	eFluor450	eBioscience	1/100
Mouse tetramer-SIINFEKL	NA	APC	Provided by NIH tetramer core facility	1/1000
CD25	PC61	PE-dazzle	BioLegend	1/50
B220	RA3-6B2	APC-Cy7	BioLegend	1/100
TCF-1/7	C63D9	PB	Cell Signaling	1/50
T-bet	4B10	BV786	BioLegend	1/50
EOMES	W17001A	PE	BioLegend	1/50
Foxp3	MF14	AF700	BioLegend	1/50
Lag3	C9B7W	PE	Biosciences	1/100
Cd62l	MEL-14	BV786	Biosciences	1/100

TotalSeq™-C0096 anti-mouse CD45 Antibody	30-F11	NA	BioLegend	1/100
CD3	17A2	PE	BioLegend	1/100
PDL1	10F.9G2	BV421	BioLegend	1/100
Ly6G	1A8	BUV737	BioLegend	1/100
MHC-II	M5/114.15.2	BV786	BioLegend	1/100
F4/80	BM8	FITC	BioLegend	1/100
CD11c	N418	PE-Cy7	BioLegend	1/200
MRC1	C068C2	AF647	BioLegend	1/100
CD86	GL-1	APC-Cy7	BioLegend	1/100
NKp46	29A1.4	PE	BioLegend	1/100

Supplementary table 6. List of antibodies employed for flow cytometry analysis.

Target	Conj	Host	Reactivity	Supplier	Cat number	Dilution
aRabbit-AF647	AF647	Donkey	Rabbit	Invitrogen	A31573	1/500
aRat-AF555	AF555	Donkey	Rat	Abcam	ab150154	1/500
CD4	/	Rat	Mouse	BioLegend	100506	1/100
CD8	/	Rat	Mouse	eBioscience	14-019-582	1/100
TRP2 (anti-DCT)	/	Rabbit	Human/Mouse	Antibodies.com	a98674	1/100

Supplementary table 7. List of antibodies employed for IF analysis.
