# Supplementary material for: In vivo armed macrophages curb liver metastasis through tumor-reactive T-cell rejuvenation
Source: Nat Commun. 2025 Apr 11;16:3471. doi: 10.1038/s41467-025-58369-2 (PMC11992024; doi:10.1038/s41467-025-58369-2)
Supplement: Supplementary file 2 — Description of Additional Supplementary Files [file 41467_2025_58369_MOESM2_ESM.pdf]

Description of Additional Supplementary Files:

Supplementary Data 1: Top markers in tumor APC populations in mice bearing MC38.OVA LM.p-value calculated by Wilcoxon Rank Sum test. Adjusted p-value is calculated based on bonferroni correction.

Supplementary Data 2: Top markers in liver T and NK population in mice bearing MC38.OVA LM. p-value calculated by Wilcoxon Rank Sum test. Adjusted p-value is calculated based on bonferroni correction.

Supplementary Data 3: Top markers in tumor APC population in mice bearing AKTPF LM. p-value calculated by Wilcoxon Rank Sum test. Adjusted p-value is calculated based on bonferroni correction.

Supplementary Data 4: Top markers in liver T and NK population in mice bearing AKTPF LM. p-value calculated by Wilcoxon Rank Sum test. Adjusted p-value is calculated based on bonferroni correction.

Supplementary Data 5: List of predicted interaction between CD4,CD8,TAMs and DCs in TA33.Combo vs TA33 treated mice. p_values were calculated employing the MultiNicheNet analysis package.

Supplementary Data 6: Transcript distance matrix normalised

Supplementary Data 7: List of human genes in the IIT signature.
