# Supplementary material for: In vivo armed macrophages curb liver metastasis through tumor-reactive T-cell rejuvenation
Source: Nat Commun. 2025 Apr 11;16:3471. doi: 10.1038/s41467-025-58369-2 (PMC11992024; doi:10.1038/s41467-025-58369-2)
Supplement: Supplementary file 10 — Reporting Summary [file 41467_2025_58369_MOESM10_ESM.pdf]

nature portfolio

Corresponding author(s): Mario Leonardo Squadrito

Last updated by author(s): 27/02/2025

Reporting Summary

Nature Portfolio wishes to improve the reproducibility of the work that we publish. This form provides structure for consistency and transparency in reporting. For further information on Nature Portfolio policies, see our Editorial Policies and the Editorial Policy Checklist.

Statistics

For all statistical analyses, confirm that the following items are present in the figure legend, table legend, main text, or Methods section.

n/a	Confirmed	
☐	☒	The exact sample size (*n*) for each experimental group/condition, given as a discrete number and unit of measurement
☐	☒	A statement on whether measurements were taken from distinct samples or whether the same sample was measured repeatedly
☐	☒	The statistical test(s) used AND whether they are one- or two-sided<br>*Only common tests should be described solely by name; describe more complex techniques in the Methods section.*
☒	☐	A description of all covariates tested
☐	☒	A description of any assumptions or corrections, such as tests of normality and adjustment for multiple comparisons
☐	☒	A full description of the statistical parameters including central tendency (e.g. means) or other basic estimates (e.g. regression coefficient) AND variation (e.g. standard deviation) or associated estimates of uncertainty (e.g. confidence intervals)
☐	☒	For null hypothesis testing, the test statistic (e.g. *F*, *t*, *r*) with confidence intervals, effect sizes, degrees of freedom and *P* value noted<br>*Give P values as exact values whenever suitable.*
☒	☐	For Bayesian analysis, information on the choice of priors and Markov chain Monte Carlo settings
☒	☐	For hierarchical and complex designs, identification of the appropriate level for tests and full reporting of outcomes
☐	☒	Estimates of effect sizes (e.g. Cohen's *d*, Pearson's *r*), indicating how they were calculated

Our web collection on statistics for biologists contains articles on many of the points above.

Software and code

Policy information about availability of computer code

Data collection	No software was used
Data analysis	All statistical analyses were performed with GraphPad prism or using R version 4.1.2 (http://www.R-project. org/).<br>RNA sequencing was analysed in R (http://www.R-project.org/) and processed with the Seurat package (http://satijalab.org/seurat v5.0.1).

For manuscripts utilizing custom algorithms or software that are central to the research but not yet described in published literature, software must be made available to editors and reviewers. We strongly encourage code deposition in a community repository (e.g. GitHub). See the Nature Portfolio guidelines for submitting code & software for further information.

Data

Policy information about availability of data

All manuscripts must include a data availability statement. This statement should provide the following information, where applicable:

- Accession codes, unique identifiers, or web links for publicly available datasets
- A description of any restrictions on data availability
- For clinical datasets or third party data, please ensure that the statement adheres to our policy

The MERFISH, single-cell RNA sequencing, and bulk RNA sequencing data have been deposited in the GEO repository under the accession number GSE273615. Additionally, the whole exome sequencing data have been uploaded to the ENA portal with the accession number PRJEB78386.

Research involving human participants, their data, or biological material

Policy information about studies with human participants or human data. See also policy information about sex, gender (identity/presentation), and sexual orientation and race, ethnicity and racism.

Reporting on sex and gender	*Use the terms sex (biological attribute) and gender (shaped by social and cultural circumstances) carefully in order to avoid confusing both terms. Indicate if findings apply to only one sex or gender; describe whether sex and gender were considered in study design; whether sex and/or gender was determined based on self-reporting or assigned and methods used. Provide in the source data disaggregated sex and gender data, where this information has been collected, and if consent has been obtained for sharing of individual-level data; provide overall numbers in this Reporting Summary. Please state if this information has not been collected. Report sex- and gender-based analyses where performed, justify reasons for lack of sex- and gender-based analysis.*
Reporting on race, ethnicity, or other socially relevant groupings	*Please specify the socially constructed or socially relevant categorization variable(s) used in your manuscript and explain why they were used. Please note that such variables should not be used as proxies for other socially constructed/relevant variables (for example, race or ethnicity should not be used as a proxy for socioeconomic status). Provide clear definitions of the relevant terms used, how they were provided (by the participants/respondents, the researchers, or third parties), and the method(s) used to classify people into the different categories (e.g. self-report, census or administrative data, social media data, etc.) Please provide details about how you controlled for confounding variables in your analyses.*
Population characteristics	*Describe the covariate-relevant population characteristics of the human research participants (e.g. age, genotypic information, past and current diagnosis and treatment categories). If you filled out the behavioural & social sciences study design questions and have nothing to add here, write "See above."*
Recruitment	*Describe how participants were recruited. Outline any potential self-selection bias or other biases that may be present and how these are likely to impact results.*
Ethics oversight	*Identify the organization(s) that approved the study protocol.*

Note that full information on the approval of the study protocol must also be provided in the manuscript.

Field-specific reporting

Please select the one below that is the best fit for your research. If you are not sure, read the appropriate sections before making your selection.

- [x] Life sciences
- [ ] Behavioural & social sciences
- [ ] Ecological, evolutionary & environmental sciences

For a reference copy of the document with all sections, see nature.com/documents/nr-reporting-summary-flat.pdf

Life sciences study design

All studies must disclose on these points even when the disclosure is negative.

Sample size	We used 5 to 13 mice per group, depending on tumor variability known from previous experiments, the chosen readout, and the number of experimental groups. Sample size was determined based on expected effect size, standard deviation, and statistical power considerations, ensuring a balance between robust statistical analysis and ethical animal use. Power calculations were performed to estimate the minimum number of animals needed to detect significant differences while accounting for tumor growth variability.
Data exclusions	No data points were excluded from the study. Only when technical errors were present, i.e. coagulation of blood, misplacement of tumors, or similar technical issues data points were excluded before experiment termination and data analysis.
Replication	Reproducibility of findings were ensured by the appropriate choice of cohort size per experimental group as well as confirmation of findings in different tumor models.
Randomization	In this study, mice were randomly allocated to experimental groups ensuring equal distribution regarding order of tumor placement and LV application as well as housing cage and lineage. Of note, in this study tumor burden in individual mice was accessed, blindly, only after allocation into distinct treatment cohorts , thus, ensuring unbiased randomization. To note that we employed preferentially cages with at least four mice, and all groups were (i.e. each mouse belonging to a different group) cohabited in the cage.
Blinding	If possible, data acquisition was performed blindly. In MRI experiments data acquisition was performed by investigator who were unaware of group belongings. For experiments where tumor growth is reported as weight, i.e. using a lab analytical balance, data acquisition was performed by investigators who were aware of group belonging for each mouse, however measurements were always performed using the same parameters for all experimental cohorts, hence avoiding any bias.

Reporting for specific materials, systems and methods

We require information from authors about some types of materials, experimental systems and methods used in many studies. Here, indicate whether each material, system or method listed is relevant to your study. If you are not sure if a list item applies to your research, read the appropriate section before selecting a response.

Materials & experimental systems

n/a	Involved in the study
☐	☒ Antibodies
☐	☒ Eukaryotic cell lines
☒	☐ Palaeontology and archaeology
☐	☒ Animals and other organisms
☐	☒ Clinical data
☒	☐ Dual use research of concern
☒	☐ Plants

Methods

n/a	Involved in the study
☒	☐ ChIP-seq
☐	☒ Flow cytometry
☒	☐ MRI-based neuroimaging

Antibodies

Antibodies used

Flow cytometry antibodies:
Target;Clone; Conjugation; Producer
CD11b M1/70 BV710 BioLegend
CD279 (PD1) 29F.1A12 PE-Cy7 BioLegend
CD4 RM4-5 BUV737 BD Horizon
CD44 IM7 BV605 BD Horizon
CD45 30-F11 BV510 BioLegend
CD62L MEL-14 BV786 eBioscience
CD8 53-6.7 FITC BD Pharmagen
Ly6c HK1.4 eFluor450 eBioscience
Mouse tetramer-SIINFEKL NA APC Provided by NIH tetramer core facility
CD25 PC61 PE-dazzle BioLegend
B220 RA3-6B2 APC-Cy7 BioLegend
TCF-1/7 C63D9 PB Cell Signaling
T-bet 4B10 BV786 BioLegend
EOMES W17001A PE BioLegend
Foxp3 MF14 AF700 BioLegend
Lag3 C9B7W PE Biosciences
Cd62l MEL-14 BV786 Biosciences
CD3 17A2 PE BioLegend
PDL1 10F.9G2 BV421 BioLegend
Ly6G 1A8 BUV737 BioLegend
MHC-II M5/114.15.2 BV786 BioLegend
F4/80 BM8 FITC BioLegend
CD11c N418 PE-Cy7 BioLegend
MRC1 C068C2 AF647 BioLegend
CD86 GL-1 APC-Cy7 BioLegend
NKp46 29A1.4 PE BioLegend

TotalSeq-C0096 anti-mouse CD45 Antibody 30-F11 NA BioLegend
TotalSeq-C0301 anti-mouse Hashtag 1 Antibody, M142; 30-F11 (biolegend.com)
TotalSeq-C0302 anti-mouse Hashtag 2 Antibody, M142; 30-F11 (biolegend.com)
TotalSeq-C0303 anti-mouse Hashtag 3 Antibody, M142; 30-F11 (biolegend.com)
TotalSeq-C0182 anti-mouse CD3 Antibody, CD3, 17A2 (biolegend.com)

Imunofluorescence antibodies:
aRabbit-AF647 AF647 Donkey Rabbit Invitrogen A31573
aRat-AF555 AF555 Donkey Rat Abcam ab150154
CD4 / Rat Mouse BioLegend 100506
CD8 / Rat Mouse eBioscience 14-019-582
TRP2 (anti-DCT) / Rabbit Human/Mouse Antibodies.com a98674

In vivo antibodies:
In-vivoMAB anti-mouse PD-1 (BioXCell, catalog number:BE0146)
In-vivoMAB anti-mouse CD4 (BioXCell, catalog number:BE0003-1)
In-vivoMAB anti-mouse CD8a (BioXCell, catalog number:BE0004-1)
In-vivoMAB anti-mouse NK 1.1 (BioXCell, catalog number:BE0036).

Validation	All antibodies in this study are commonly used, and have been validated by previous studies.

Eukaryotic cell lines

Policy information about cell lines and Sex and Gender in Research

Cell line source(s)	HEK293T cells were obtained from repository ATCC.<br>MC38 and B16-F10 were obtained by Amgen.<br>MC38.OVA were generated by transduction of MC38 with a PGK.OVA lentiviral vector. Vector copy number and OVA presentation/ gene expression were used to confirm OVA expression by the cell line.<br>AKTPF organoids were obtained from Masanobu Lab. AKTPF cells were generated by passing the cells through passages in immunodeficient NSG and immunocompetent C57BL6 mice.
Authentication	None of the cells were authenticated by our group, but have been obtained as described above from reliable sources. However, whole exosome sequencing and RNA sequencing analysis confirmed the identity of AKPTF cells.
Mycoplasma contamination	Cells were tested routinely (once per month) for mycoplasma contamination and resulted always negative.
Commonly misidentified lines (See ICLAC register)	None of the cells used in this study have been misidentified according to the ICLAC register.

Animals and other research organisms

Policy information about studies involving animals; ARRIVE guidelines recommended for reporting animal research, and Sex and Gender in Research

Laboratory animals	In this study we used C57BL6n mice obtained from Charles River Laboratories, Italy.
Wild animals	The study did not involve the use of wild animals.
Reporting on sex	This study involved the use of both mouse genders. However, we employed mainly female mice, since are less aggressive minimizing stress-related variables that can affect experimental outcomes.
Field-collected samples	Samples were not collected from the field.
Ethics oversight	All experiments and procedures were performed according to protocols approved by the Institutional Animal Care and Use Committee (IACUC) at San Raffaele Hospital animal facilities (IACUC number: 1098, 1108, 1227, 1383) and authorized by the Italian Ministry of Health and local authorities according to the Italian law.

Note that full information on the approval of the study protocol must also be provided in the manuscript.

Clinical data

Policy information about clinical studies

All manuscripts should comply with the ICMJEguidelines for publication of clinical research and a completedCONSORT checklist must be included with all submissions.

Clinical trial registration	*Provide the trial registration number from ClinicalTrials.gov or an equivalent agency.*
Study protocol	*Note where the full trial protocol can be accessed OR if not available, explain why.*
Data collection	*Describe the settings and locales of data collection, noting the time periods of recruitment and data collection.*
Outcomes	*Describe how you pre-defined primary and secondary outcome measures and how you assessed these measures.*

Plants

Seed stocks	*Report on the source of all seed stocks or other plant material used. If applicable, state the seed stock centre and catalogue number. If plant specimens were collected from the field, describe the collection location, date and sampling procedures.*
Novel plant genotypes	*Describe the methods by which all novel plant genotypes were produced. This includes those generated by transgenic approaches, gene editing, chemical/radiation-based mutagenesis and hybridization. For transgenic lines, describe the transformation method, the number of independent lines analyzed and the generation upon which experiments were performed. For gene-edited lines, describe the editor used, the endogenous sequence targeted for editing, the targeting guide RNA sequence (if applicable) and how the editor was applied.*
Authentication	*Describe any authentication procedures for each seed stock used or novel genotype generated. Describe any experiments used to assess the effect of a mutation and, where applicable, how potential secondary effects (e.g. second site T-DNA insertions, mosiacism, off-target gene editing) were examined.*

Flow Cytometry

Plots

Confirm that:

- [ ] The axis labels state the marker and fluorochrome used (e.g. CD4-FITC).
- [x] The axis scales are clearly visible. Include numbers along axes only for bottom left plot of group (a 'group' is an analysis of identical markers).
- [x] All plots are contour plots with outliers or pseudocolor plots.
- [x] A numerical value for number of cells or percentage (with statistics) is provided.

Methodology

Sample preparation	For endpoint analysis, mice were euthanized by cervical dislocation. The liver was perfused by injecting 10 mL of PBS containing 5 mM EDTA (Invitrogen) through the inferior vena cava and cutting the portal vein to allow exiting of the solution to most circulating blood cells from the liver. When FC analysis was performed, 10 mL of IMDM (Corning) containing 0.35 mg/mL collagenase (Sigma-Aldrich) was injected through the inferior vena cava.
Instrument	Samples were acquired by using either a FACSCanto II or a FACSymphony™ A5 Cell Analyzer (BD Biosciences). For fluorescence activated cell sorting a BD FACSAria Fusion was used.
Software	FC analysis were performed using FlowJo version 10.8.1.
Cell population abundance	Cell sorted populations were sorted based on live dead, physical parameters and surface antibody staining. Cells were manually counted and identity was confirmed by FC and single cell RNA sequencing.
Gating strategy	Viability of cells was assessed by using either 7AAD nuclear staining or LIVE/DEAD™ Fixable Blue Dead Cell Stain Kit (Invitrogen) according to manufacturer's recommendation for fixed samples. Upon single cell dissociation, to prevent unspecific staining through binding of the FC receptor, we added to the cells Fc Block (BD Pharmagen). For membrane bound antigens, samples were stained for 15 minutes on ice. For staining of intracellular proteins, cells were fixed, permeabilized and stained using the True-Nuclear™ Transcription Factor Buffer Set (BioLegend) according to manufacturer's recommendation. For the staining of TCRs specific for the SIINFEKL peptide loaded on MHC class I (H2kb), samples were stained with an SIINFEKL-loaded MHC class I tetramer for 15 minutes on ice.<br>Cells were gated by identifying single cells based on physical parameters (FSC-H vs. FSC-A and SSC-A vs. FSC-A). Viable cells were identified as viability dye negative cells. CD45+ viable cells were divided as follow: For the lymphoid cell characterization: B cells (CD45+ CD11b- B220+), CD4 T cells (CD45+ CD11b- B220- CD4+), CD8 T cells (CD45+ CD11b- B220- CD8+), OVA-specific CD8 T cells (CD45+ CD11b- B220- CD8+, SIINFEKL-MHC+). Whitin CD8+ T cells or OVA specific T cells, we defined TEX as EOMES+ PD1high and PEX as TBET+ PD1int. For the myeloid cell characterization, we gated myeloid cells as follow: KCs (CD45+ CD11b+ F4/80high), granulocytes (CD45+ CD11b+ Ly6ghigh), DC (CD45+CD11b+Ly6g-CD11c+), TAMs (CD45+CD11b+Ly6g- CD11c-F4/80+), monocytes (CD45+CD11b+Ly6g- CD11c- F4/80-).

- [x] Tick this box to confirm that a figure exemplifying the gating strategy is provided in the Supplementary Information.
